# Multidimensional nutritional assessment in Crohn’s disease: Cross-sectional comparison of active disease and remission

**DOI:** 10.1371/journal.pone.0352372

**Published:** 2026-09-03

**Authors:** Aditi Sarker, Chanchal Kumar Ghosh, Prodipta Chowdhury

**Affiliations:** 1 Department of Gastroenterology, Bangladesh Medical University, Shahbagh, Dhaka, Bangladesh; 2 Department of Endocrinology, Bangladesh Medical University, Shahbagh, Dhaka, Bangladesh; University of Diyala College of Medicine, IRAQ

## Abstract

Malnutrition is a frequent complication of Crohn’s disease (CD), but multidimensional nutritional data from South Asian populations remain limited. This cross-sectional study compared nutritional status between active disease and remission among 127 South Asian adults with CD attending a tertiary-care center in Dhaka, Bangladesh. Disease activity was assessed using the Crohn’s Disease Activity Index (CDAI), Simple Endoscopic Score for Crohn’s Disease (SES-CD), and magnetic resonance enterography (MRE). Nutritional assessment included body mass index (BMI), mid-upper arm circumference (MUAC), calf circumference (CC), triceps skinfold thickness (TSF), mid-arm muscle circumference (MAMC), the Mini Nutritional Assessment (MNA), and biochemical measurements. Protein-energy malnutrition was operationalized using the Global Leadership Initiative on Malnutrition (GLIM) framework, with BMI < 18.5 kg/m^2^ as the available phenotypic criterion and CD-related inflammatory and gastrointestinal burden as the etiologic criterion. Of 127 participants, 63 had active disease, and 64 were in remission. Overall, 60 participants (47.2%) met the study definition of GLIM malnutrition, with a substantially higher frequency in active disease than in remission (81.0% vs. 14.1%, P < 0.001). Participants with active disease had significantly lower BMI, MUAC, CC, sex-stratified TSF, MAMC, and MNA scores (all P < 0.001). Hemoglobin, serum iron, albumin, and zinc concentrations were also lower in active disease (all P < 0.001), whereas folate (P = 0.411) and vitamin B12 (P = 0.051) did not differ significantly. BMI correlated positively with MUAC, CC, TSF, MAMC, and MNA score (r_s_ = 0.366–0.967; all P < 0.001), with the weakest association observed for MAMC, while correlations with biochemical markers were weaker and were generally not statistically significant after subgroup stratification. The objective disease-activity sensitivity analysis based on SES-CD and/or MRE produced the same active and remission classification as the primary analysis (100% agreement; Cohen’s κ = 1.000). These findings demonstrate a substantial burden of protein-energy malnutrition in Bangladeshi adults with CD, particularly during active disease, while also showing that nutritional impairment may persist during remission. Simple anthropometric measures provide useful information on physical tissue depletion, but biochemical abnormalities should be interpreted alongside disease activity because markers such as albumin and zinc may be influenced by systemic inflammation in addition to nutritional status. A multidimensional approach combining anthropometric assessment with targeted biochemical evaluation may therefore provide a more comprehensive assessment of nutritional status in CD.

## 1. Introduction

Crohn’s disease (CD) is a chronic, relapsing inflammatory bowel disease that can involve any part of the gastrointestinal tract. Malnutrition is a common but often underrecognized complication of CD and may influence functional status, treatment tolerance, quality of life, and overall clinical outcome alongside control of intestinal inflammation [1,2]. Reported rates of malnutrition vary widely according to disease activity, clinical setting, and the method used for nutritional assessment, but the burden is consistently greater during active disease than during remission [2,3].

Nutritional impairment in CD is multifactorial. Reduced food intake, malabsorption, enteric nutrient loss, increased metabolic demand, and cytokine-mediated catabolism may contribute to loss of body mass and tissue stores [4,5]. Westernized dietary exposures may further influence intestinal and metabolic inflammation; high fructose intake has been associated with worsening experimental colitis, while alcohol consumption has been linked to adverse disease outcomes in IBD and may contribute to metabolic dysfunction and systemic inflammation [6–8]. Patients with CD may continue to experience nutritional and functional deficits even during prolonged remission despite apparently adequate macronutrient intake, so deficiencies of iron, folate, vitamin B12, zinc, and other micronutrients may occur during both active disease and remission, highlighting the need for routine nutritional assessment beyond symptomatic disease alone [4,5,9]. Biochemical abnormalities, however, do not always represent nutrient depletion alone. Inflammatory activity, intestinal blood loss, disease location, treatment exposure, and supplementation can influence hemoglobin, iron, albumin, and zinc concentrations, while albumin and zinc may also change as part of the acute-phase response. Nutritional assessment in CD therefore requires interpreting biochemical findings alongside measures of physical nutritional status, rather than relying on a single laboratory marker.

No single measure captures all dimensions of nutritional impairment in CD [3]. Anthropometric indices such as body mass index (BMI), mid-upper arm circumference (MUAC), calf circumference (CC), triceps skinfold thickness (TSF), and mid-arm muscle circumference (MAMC) provide practical information about body mass and tissue stores, particularly where advanced body-composition techniques are not readily available [3,10]. The Mini Nutritional Assessment (MNA) can provide additional information, although the MNA was developed primarily for older adults and is not specific to Crohn’s disease; it was included in the present study as a complementary measure of nutritional status rather than as the primary diagnostic criterion for malnutrition [11]. The Global Leadership Initiative on Malnutrition (GLIM) framework, introduced in 2019, provides a standardized approach to diagnosing adult protein-energy malnutrition across diverse clinical populations and has increasingly been applied in patients with CD [12,13]. But these approaches are complementary: anthropometry and GLIM address physical or protein-energy depletion, whereas targeted biochemical testing is needed to identify micronutrient abnormalities that may occur independently of body mass.

The limitations of BMI are particularly relevant in South Asian populations. A characteristic thin-fat phenotype, with relatively greater central adiposity and metabolic risk at lower BMI, can result in apparently acceptable body weight despite unfavorable body composition or reduced lean tissue [14]. Studies from India have also demonstrated altered body composition in patients with CD across active and remission phases [15]. The growing burden of inflammatory bowel disease across regions undergoing rapid epidemiological transition further increases the need for locally grounded evidence [16]. Thus, BMI alone may fail to identify some forms of muscle or tissue depletion in this population. In the present study, the etiologic criterion was based on Crohn’s disease-related inflammatory and gastrointestinal burden, and BMI < 18.5 kg/m² was used as the phenotypic GLIM criterion for low BMI, consistent with the underweight threshold used in Asian adults; however, this threshold does not overcome the inherent inability of BMI to distinguish fat mass from lean mass. For this reason, BMI was interpreted alongside MUAC, CC, TSF, and MAMC, although these measures were not used as formal GLIM diagnostic criteria because a prospectively specified, validated regional cutoff for reduced muscle mass was unavailable.

Despite the clinical importance of these issues, multidimensional nutritional data from South Asian adults with CD remain limited. Previous studies from other populations have shown poorer nutritional status during active disease [17], and regional evidence supports the importance of body-composition assessment [14,15], but few studies have compared active disease and remission while integrating anthropometry, a structured nutritional assessment, GLIM-based classification, biochemical measurements, and complementary clinical, endoscopic, and radiological assessment of disease activity. This distinction is important because symptom-based activity may not always correspond to objective intestinal inflammation. Accordingly, this study aimed to compare nutritional status between adults with active CD and those in remission in a tertiary-care setting in Dhaka, Bangladesh, using a multidimensional assessment that included anthropometric measures, the full MNA, GLIM-based protein-energy malnutrition classification, and selected biochemical markers. We evaluated disease activity using the Crohn’s Disease Activity Index, the Simple Endoscopic Score for Crohn’s Disease, and magnetic resonance enterography [18–21], and conducted an objective sensitivity analysis based on endoscopic and/or radiological activity to assess whether the nutritional findings depended on clinical symptoms alone.

## 2. Methods

### 2.1. Ethics statement

We conducted this study in compliance with the Declaration of Helsinki. The study’s protocol was reviewed and approved by the Institutional Review Board (IRB) of Bangladesh Medical University, Shahbagh, Dhaka (approval no. 4764; 11 Jan 2024), and written informed consent was obtained from all participants. This study was reported in accordance with the Strengthening the Reporting of Observational Studies in Epidemiology (STROBE) statement for cross-sectional studies; the completed checklist is provided as S1 File.

### 2.2. Study design and participants

This cross-sectional observational study was conducted among patients attending the inpatient and outpatient gastroenterology services and the inflammatory bowel disease clinic of a tertiary-care hospital in Dhaka, Bangladesh. Adults aged 18 years or older with previously or newly diagnosed Crohn’s disease were consecutively assessed from 1 February 2024–31 January 2025, following ethics approval. Sex was self-reported and recorded as a biological variable at enrolment. Crohn’s disease was diagnosed based on compatible clinical, endoscopic, histological, and imaging findings.

**Fig 1:**
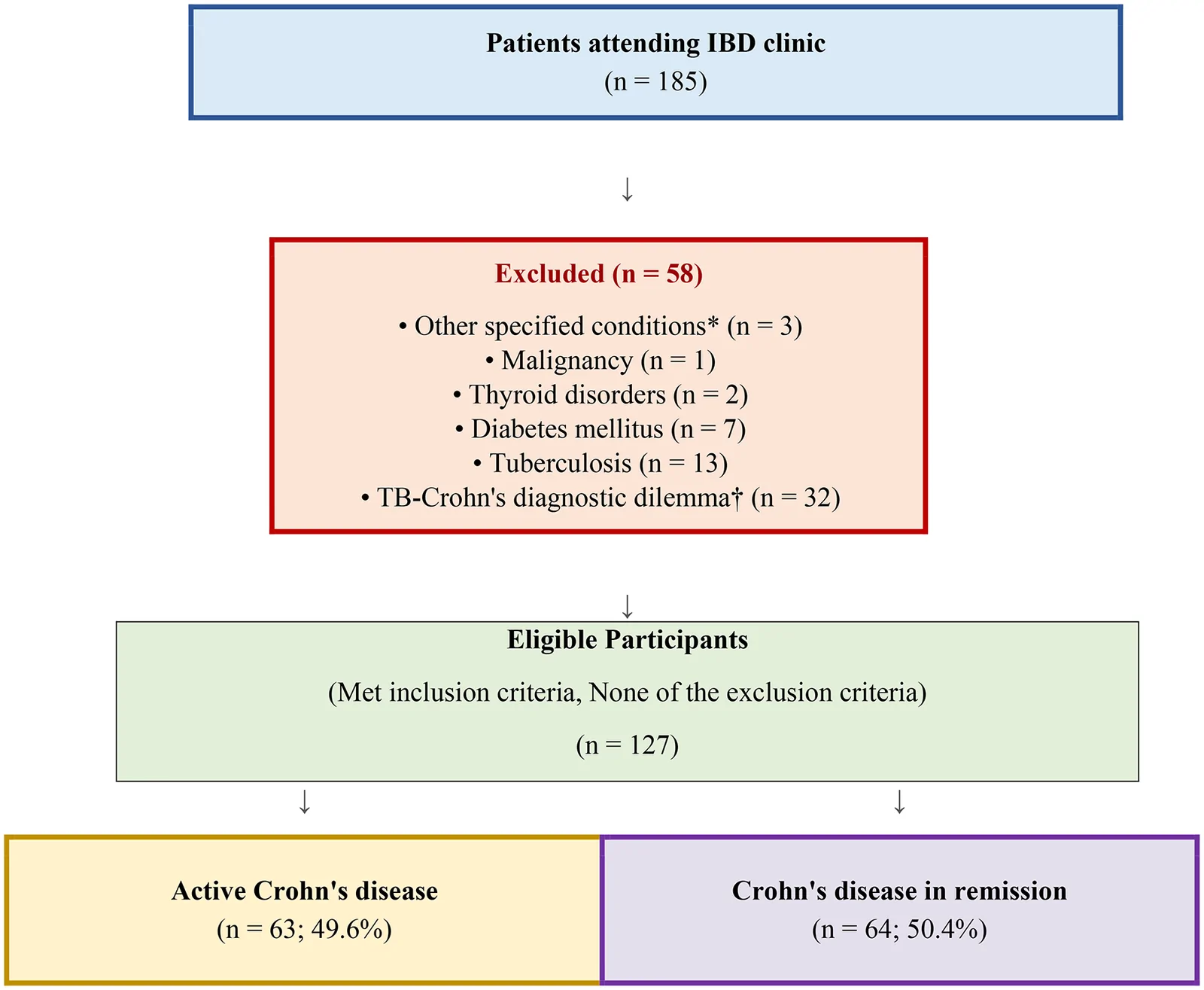
Participant flow diagram (*Other specified conditions included chronic liver disease, nephrotic syndrome, chronic pancreatitis, advanced cardiopulmonary disease, and chronic kidney disease. †TB-Crohn’s diagnostic dilemma refers to cases in which intestinal tuberculosis and Crohn’s disease could not be differentiated with sufficient diagnostic confidence.).

We assessed 185 CD patients for eligibility. Patients were excluded if they were pregnant or had conditions that could independently affect nutritional status, including chronic liver disease, nephrotic syndrome, chronic pancreatitis, diabetes mellitus, tuberculosis, thyroid disorders, advanced cardiopulmonary disease, chronic kidney disease, malignancy, or unresolved diagnostic uncertainty between intestinal tuberculosis and Crohn’s disease. A detailed breakdown of exclusions is presented in Fig 1. The remaining 127 participants were included in the study, comprising 63 with active Crohn’s disease and 64 in remission.

These exclusion criteria were prespecified to minimise confounding from conditions that could independently cause weight loss, systemic wasting, or abnormalities in biochemical nutritional markers. Exclusion of confirmed tuberculosis and cases with unresolved tuberculosis–Crohn’s disease diagnostic uncertainty was particularly important because intestinal tuberculosis may closely resemble Crohn’s disease. However, these exclusions may reduce the generalisability of the findings to an unselected tertiary-care population. Written informed consent was obtained from all participants before study procedures. All 127 enrolled participants were included in the final analysis, and no missing observations were identified in the variables analysed.

### 2.3. Disease activity assessment

Disease activity was evaluated using three complementary modalities: the Crohn’s Disease Activity Index (CDAI) for clinical activity, the Simple Endoscopic Score for Crohn’s Disease (SES-CD) for endoscopic activity, and magnetic resonance enterography (MRE) for radiological activity [18–21].

**Fig 2:**
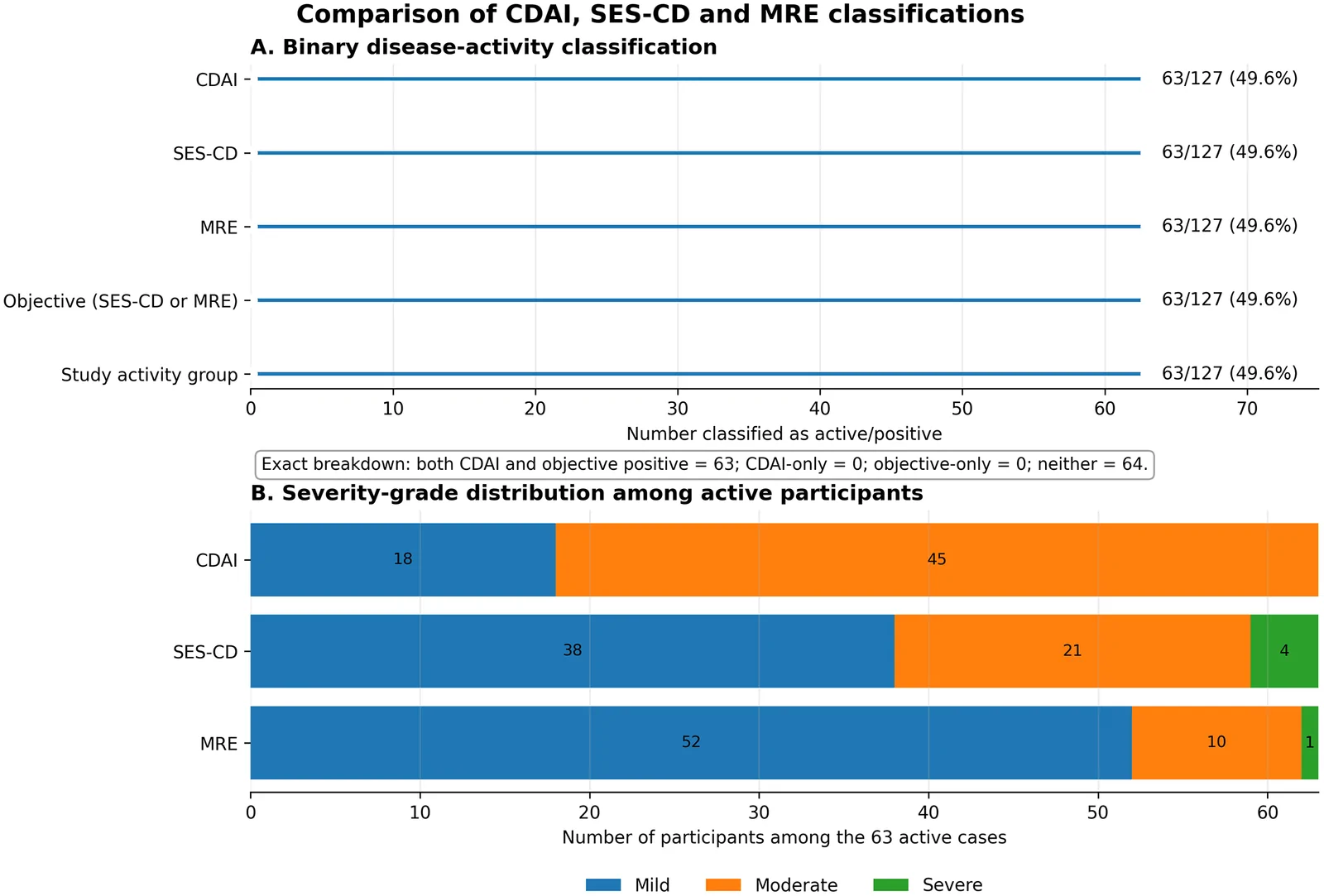
Comparison of CDAI, SES-CD, and MRE classifications. Panel A shows identical binary active-disease classification across the three modalities, the objective composite definition, and the study grouping. Panel B shows the distributions of severity grades among the 63 active participants. Objective activity was defined as activity on SES-CD and/or MRE.

For the primary analysis, participants were classified as having active disease when activity was identified on at least one of the three assessments, whereas remission required the absence of activity across all three modalities. To examine the possibility of symptom-driven misclassification, a sensitivity analysis defined active disease strictly by objective evidence of inflammation on either SES-CD or MRE, irrespective of CDAI. Binary agreement between the original disease-activity classification and the objective definition was assessed using cross-tabulation, percentage agreement, and Cohen’s κ. Comparisons of ordinal severity categories among CDAI, SES-CD, and MRE were assessed using Spearman’s rank correlation coefficient. The results of these comparisons are presented in Fig 2.

### 2.4. Nutritional assessment

Nutritional status was evaluated using anthropometry, the Mini Nutritional Assessment (MNA), and biochemical measurements. Height was measured to the nearest 0.1 cm using a wall-mounted stadiometer, and body weight was measured with minimal clothing using a calibrated electronic scale. BMI was calculated as weight in kilograms divided by height in meters squared. Mid-upper arm circumference (MUAC) and calf circumference (CC) were measured with a non-stretch tape to the nearest 0.1 cm. Triceps skinfold thickness (TSF) was measured at the midpoint between the acromion and olecranon using skinfold callipers, and mid-arm muscle circumference (MAMC) was calculated as MUAC (cm) − [π × TSF (mm) / 10]. Measurements were obtained on the non-dominant arm using a standardised technique. The full MNA (0–30 points) was administered as specified in the approved protocol; scores <17 indicate malnutrition, 17-23.5 indicate a risk of malnutrition, and ≥24 indicate normal nutritional status. Because the MNA was not developed specifically for younger adults with CD, it was interpreted only as a complementary assessment.

### 2.5. Definition of Malnutrition

Protein-energy malnutrition was operationalised using the Global Leadership Initiative on Malnutrition (GLIM) framework [12]. The phenotypic criterion used in this dataset was low BMI, defined as BMI < 18.5 kg/m²; the etiologic criterion was fulfilled by CD-related inflammatory and gastrointestinal burden. Although MUAC, CC, TSF, and MAMC were measured, they were not used to trigger the GLIM diagnosis because a prospectively specified, validated regional cutoff for reduced muscle mass was not available. Weight-loss history was incomplete and was also not used as a diagnostic trigger. GLIM was therefore analysed dichotomously as malnutrition present or absent, without severity staging. Albumin and micronutrient concentrations were not GLIM diagnostic criteria and were analysed separately.

### 2.6. Biochemical and imaging assessment

Haemoglobin was measured using a Sysmex XN2000 six-part differential automated haematology analyser in the institutional haematology laboratory. Serum zinc was analysed using an Indiko Plus analyser, and serum vitamin B12 and folate were measured using the Alinity ci analyser in the institutional biochemistry laboratory. The remaining biochemical assays, including serum iron and albumin, were performed using the Atellica chemistry system (Siemens). Colonoscopy was performed in the institutional gastroenterology unit using Olympus CV 170 equipment, and MRE was performed in the institutional radiology department using the Magnetom Sempra system (Siemens).

### 2.7. Sample size

The required sample size was estimated from a previously reported between-group difference in BMI among patients with Crohn’s disease [22]. For comparison of two independent means with equal group sizes, the sample size for each group was calculated using the following formula [23]:


$$\begin{array}{c} \text{n }=\;[{\left({\text{Z}}_{\text{1}}-\alpha /2\;+\;{\text{Z}}_{\text{1}}-\beta \right)}^{\text{2}}\;({\sigma_{\text{1}}}^{\text{2}}\;+\;{\sigma_{\text{2}}}^{\text{2}})]\;/\;{\left(\mu_{\text{1}}\;-\;\mu_{\text{2}}\right)}^{\text{2}} \end{array}$$

Here, Z₁ ₋ α⁄2 was 1.96 for a two-sided 95% confidence level, Z₁ ₋ β was 1.645 for 95% power, μ₁ and μ2 were the reported mean BMI values in the remission and active-disease groups (21.6 and 18.8 kg/m², respectively), and σ₁ and σ2 were the corresponding standard deviations (5.0 and 3.6 kg/m², respectively). The calculation yielded a minimum of 63 participants per group. We enrolled 127 participants: 63 with active disease and 64 in remission.

### 2.8. Statistical analysis

Data were analysed using SPSS version 25.0 (IBM Corp., Armonk, NY, USA). Distributional assumptions were assessed separately within the active-disease and remission groups using the Shapiro-Wilk test together with visual inspection of histograms and Q-Q plots. Age, disease duration, BMI, MUAC, CC, TSF, MAMC, MNA, haemoglobin, serum iron, folate, vitamin B12, albumin, and zinc were analysed non-parametrically because they showed non-normality in at least one comparison group or were assessed within smaller sex-specific subgroups.

Categorical variables are reported as frequencies and percentages. Normally distributed continuous variables are reported as mean ± standard deviation (SD), whereas non-normally distributed variables are reported as median and interquartile range (IQR). Between-group categorical comparisons used the chi-square test. Normally distributed continuous variables were compared using the independent-samples Student’s t-test, and non-normally distributed variables were compared using the Mann-Whitney U test. Correlations were examined using Pearson’s correlation coefficient (r) when parametric assumptions were satisfied and Spearman’s rank correlation coefficient (rs) otherwise. Positive coefficients indicated direct associations, whereas negative coefficients indicated inverse associations.

We assessed agreement among the binary CDAI, SES-CD, MRE, objective composite, and study disease-activity classifications using cross-tabulation, percentage agreement, and Cohen’s kappa. Associations among ordinal disease-severity grades were examined using Spearman’s rank correlation. Analyses used complete observations; no data were imputed. Extreme values were checked against the source records and retained when clinically plausible. All tests were two-sided, P < 0.05 was considered statistically significant, and no adjustment for multiple comparisons was applied because the analyses were exploratory.

## 3. Results

### 3.1. Participant flow and baseline characteristics

A total of 185 patients were assessed for eligibility; 58 were excluded for prespecified reasons, and 127 participants were included in the final analysis (Fig 1). Among the analyzed participants, 63 had active Crohn’s disease, 64 were in remission, and complete data were available for all variables.

Table 1 summarizes baseline characteristics. The active-disease group contained a higher proportion of males than the remission group (65.1% vs. 46.9%, P = 0.039). Extraintestinal manifestations were more frequent in active disease than in remission (63.5% vs. 26.6%, P < 0.001). Age, residence, income, smoking status, disease duration, disease location, disease behavior, perianal disease, and relapse frequency did not differ significantly between groups.

**Table 1:** Baseline Characteristics of Study Participants (N = 127).

Characteristics		Total participants (n = 127)	Active (n = 63)	Remission (n = 64)	P value
Age (years) (Median, IQR)		32 (24, 43)	35 (24, 43)	30.5 (23.5, 41.5)	0.377 (U)
Sex	Male	71 (55.9%)	41 (65.1%)	30 (46.9%)	0.039 (C)^b^
	Female	56 (44.1%)	22 (34.9%)	34 (53.1%)	
Residence	Rural	76 (59.8%)	37 (58.7%)	39 (60.9%)	0.8 (C)
	Urban	51 (40.2%)	26 (41.3%)	25 (39.1%)	
Average monthly income	< 12,900 Taka	49 (38.6%)	22 (34.9%)	27 (42.2%)	0.572 (C)
	12,900−21,500 Taka	63 (49.6%)	32 (50.8%)	31 (48.4%)	
	> 21,500 Taka	15 (11.8%)	9 (14.3%)	6 (9.4%)	
Smoking status	Smoker	19 (15%)	11 (17.5%)	8 (12.5%)	0.433 (C)
	Non-smoker	108 (85%)	52 (82.5%)	56 (87.5%)	
Disease duration in months (Median, IQR)		48 (24, 60)	48 (24, 60)	36 (24, 60)	0.932 (U)
Disease location	Terminal ileum	42 (33.1%)	15 (23.8%)	27 (42.2%)	
	Colon	27 (21.3%)	14 (22.2%)	13 (20.3%)	
	Ileo-colon	49 (38.6%)	25 (39.7%)	24 (37.5%)	
	Isolated upper-GI	9 (7.1%)	9 (14.3%)	0	
Disease behavior	Nonstricturing, nonpenetrating	99 (78%)	44 (69.8%)	55 (85.9%)	
	Stricturing	18 (14.2%)	12 (19%)	6 (9.4%)	
	Fistulizing	5 (3.9%)	3 (4.8%)	2 (3.1%)	
	Stricturing & fistulizing	5 (3.9%)	4 (6.3%)	1(1.6%)	
Perianal disease	Yes	33 (26%)	17 (27%)	16 (25%)	0.799 (C)
	No	94 (74%)	46 (73%)	48 (75%)	
Presence of any EIM	Yes	57 (44.9%)	40 (63.5%)	17 (26.6%)	**<0.001 (C)**
	No	70 (55.1%)	23 (36.5%)	47 (73.4%)	
Relapse	Infrequent(≤1/year)	114 (89.8%)	57 (90.5%)	57 (89.1%)	0.793 (C)
	Frequent(≥2/year)	13 (10.2%)	6 (9.5%)	7 (10.9%)	

Data were expressed as median (interquartile range, IQR) for quantitative variables and frequencies (percentages) for qualitative variables. *P*-values were calculated between the active and remission groups.

EIM, Extraintestinal manifestation; C, Chi-square test; U, Mann-Whitney U test.

### 3.2. Disease-activity classification and objective sensitivity analysis

All 63 *p*articipants in the active-disease group had active findings on CDAI, SES-CD, and MRE, whereas all 64 participants in remission were inactive on all three modalities. Thus, 63 participants were positive on both CDAI and the objective composite definition, 64 were negative on both, and no CDAI-only or objective-only active cases were observed. The objective definition based on SES-CD and/or MRE reproduced the original grouping exactly (100% agreement; Cohen’s kappa = 1.000), and no participant was reclassified. Consequently, all nutritional comparisons were unchanged in the objective-only sensitivity analysis.

Although binary classification was identical, severity grading differed among the 63 active participants: CDAI classified 18 (28.6%) as mild and 45 (71.4%) as moderate; SES-CD classified 38 (60.3%) as mild, 21 (33.3%) as moderate, and 4 (6.3%) as severe; and MRE classified 52 (82.5%) as mild, 10 (15.9%) as moderate, and 1 (1.6%) as severe (Fig 2). Across the full cohort, ordinal correlations were strong for CDAI versus SES-CD (rho = 0.924), CDAI versus MRE (rho = 0.929), and SES-CD versus MRE (rho = 0.930; all P < 0.001). Within the active group, however, severity-grade correlations were weak and not statistically significant (rho = 0.240, P = 0.058; rho = 0.092, P = 0.471; and rho = 0.208, P = 0.102, respectively).

### 3.3. Anthropometric and questionnaire-based nutritional assessment

Participants with active Crohn’s disease had lower BMI, MUAC, CC, sex-stratified TSF, MAMC, and MNA scores than participants in remission (all P < 0.001; Table 2).

**Table 2:** Anthropometric and MNA assessments of study participants (N = 127).

Characteristic	Total (n = 127)	Active (n = 63)	Remission (n = 64)	P value
BMI (kg/m²)	19 (16.5, 20.6)	16.9 (15.5, 18.2)	20.4 (19.5, 21)	<0.001 (U)
MUAC (cm)	24 (21, 25)	21 (19, 22)	25 (24, 25)	<0.001 (U)
CC (cm)	31 (29, 32)	29 (28, 30)	32 (32, 32)	<0.001 (U)
TSF, male (mm)	15.6 (12.8, 23.2)	13.1 (11.85, 14.9)	22.8 (19.5, 25.8)	<0.001 (U)
TSF, female (mm)	19 (14.85, 25.65)	14.8 (13.1, 16.4)	21.75 (18.5, 27.9)	<0.001 (U)
MAMC (cm)	16.85 (15.88, 17.77)	16.26 (15.26, 17.11)	17.24 (16.47, 18.12)	<0.001 (U)
MNA score	23.5 (15.5, 25.5)	16 (14.5, 17.5)	25 (24, 25.5)	<0.001 (U)

Values are median (IQR). BMI, body mass index; MUAC, mid-upper arm circumference; CC, calf circumference; TSF, triceps skinfold thickness; MAMC, mid-arm muscle circumference; MNA, Mini Nutritional Assessment; U, Mann-Whitney U test.

### 3.4. GLIM nutritional classification

**Fig 3:**
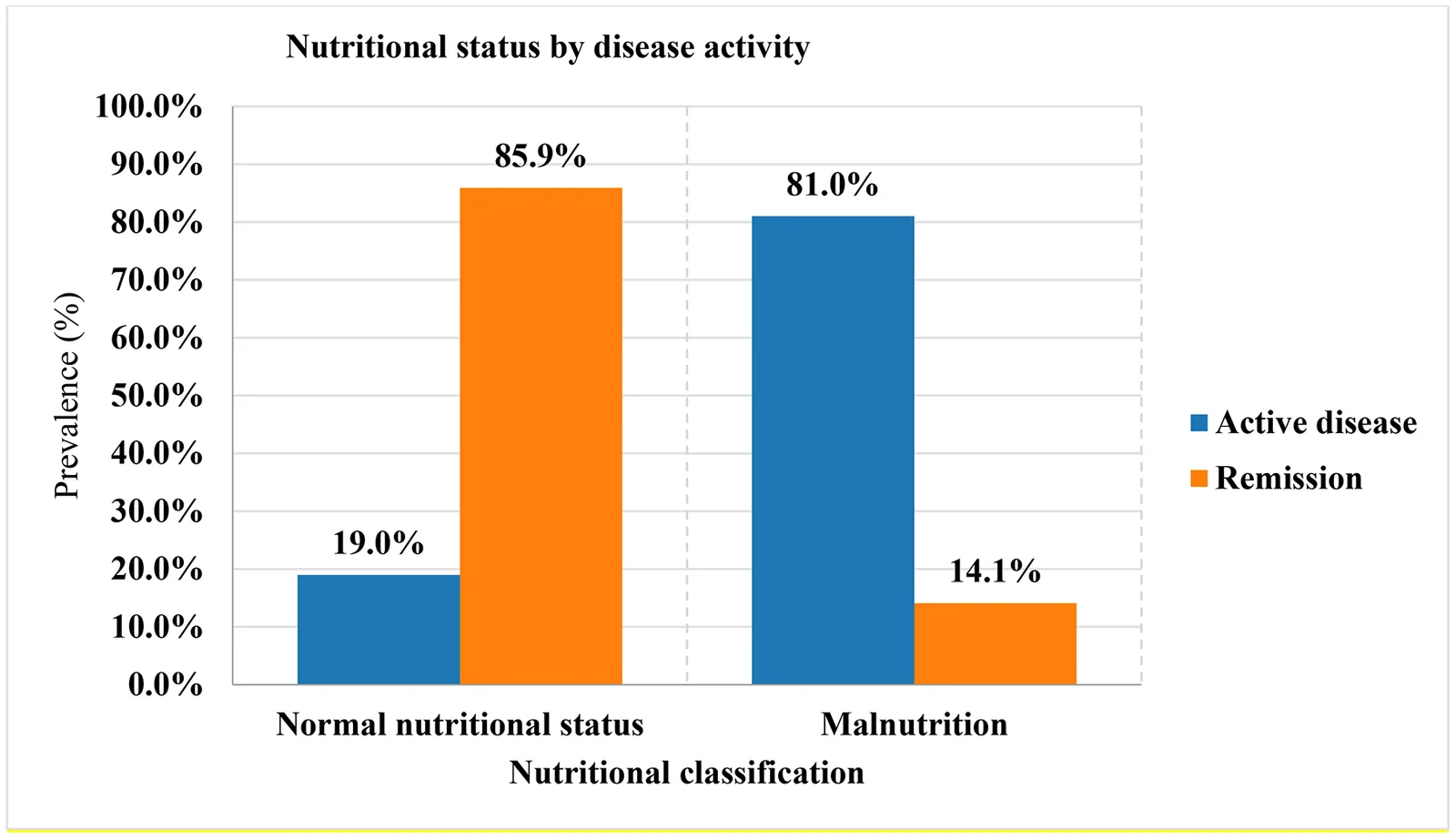
Nutritional status by disease activity. Clustered bar chart showing the prevalence of normal nutritional status and GLIM-defined malnutrition among participants with active Crohn’s disease and those in remission.

Overall, 60 of 127 participants (47.2%) met the study definition of GLIM malnutrition. Malnutrition was substantially more frequent in active disease than in remission [51/63 (81.0%) vs. 9/64 (14.1%), P < 0.001], whereas normal nutritional status was observed in 12/63 (19.0%) and 55/64 (85.9%) in active disease and remission, respectively (Fig 3).

### 3.5. Biochemical parameters

Hemoglobin concentrations were lower in active disease in both males and females (both P < 0.001). Serum iron, albumin, and zinc were also lower in active disease than in remission (all P < 0.001), whereas folate (P = 0.411) and vitamin B12 (P = 0.051) did not differ significantly (Table 3).

**Table 3:** Biochemical parameters of study participants (N = 127).

Characteristic	Total (n = 127)	Active (n = 63)	Remission (n = 64)	P value
Hemoglobin, male (g/dL)	11.3 (10.2, 12.5)	10.5 (9.5, 12.05)	12.45 (11.3, 13.2)	<0.001 (U)
Hemoglobin, female (g/dL)	10.65 (10, 11.5)	10.15 (9.4, 10.6)	11.4 (10.5, 12.2)	<0.001 (U)
Serum iron (µg/dL)	45 (32, 58)	35 (25, 45)	55 (45, 65)	<0.001 (U)
Folate (ng/mL)	6.5 (5.6, 7.7)	6.4 (4.1, 7.8)	6.5 (5.6, 7.55)	0.411 (U)
Vitamin B12 (pg/mL)	465 (306, 578)	554 (306, 630)	360 (303, 560)	0.051 (U)
Albumin (g/L)	34 (28, 40)	28 (25, 34)	37 (35.5, 41)	<0.001 (U)
Zinc (µg/dL)	65 (50, 82)	52 (45, 70)	79 (63.5, 85)	<0.001 (U)

Values are median (IQR). U, Mann-Whitney U test.

**Fig 4:**
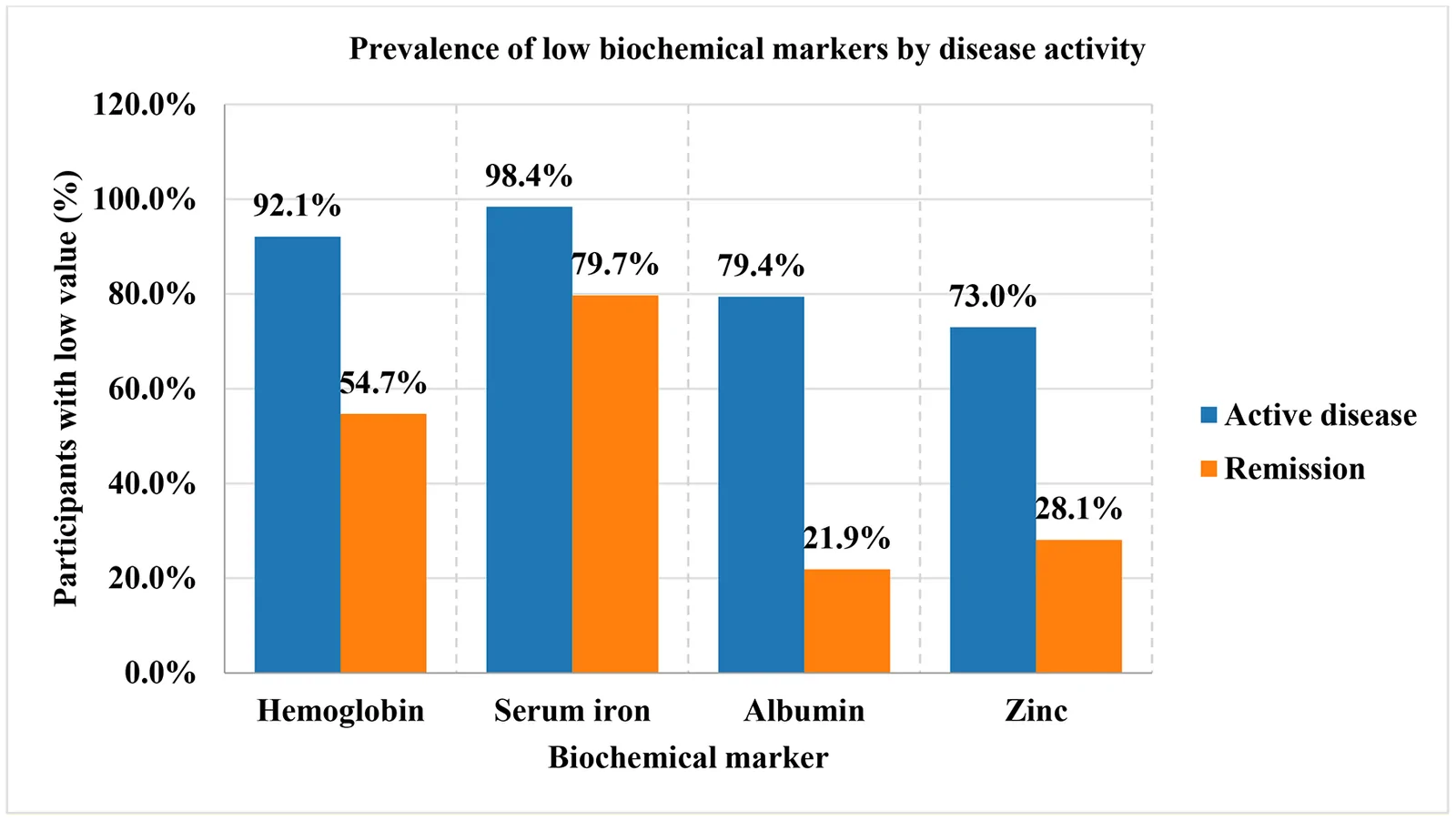
Prevalence of low biochemical markers by disease activity. Clustered bar chart showing the percentage of participants with low hemoglobin, serum iron, albumin, and zinc in active Crohn’s disease and remission.

The prevalence of low hemoglobin, serum iron, albumin, and zinc was higher in active disease than in remission: 92.1% versus 54.7% for hemoglobin (P < 0.001), 98.4% versus 79.7% for serum iron (P = 0.001), 79.4% versus 21.9% for albumin (P < 0.001), and 73.0% versus 28.1% for zinc (P < 0.001; Fig 4).

### 3.6. Correlation of BMI with anthropometric measures and MNA score

BMI showed strong to very strong positive correlations with MUAC, CC, TSF, and MNA score, while its correlation with MAMC was more modest (all P < 0.001; Table 4).

**Table 4:** Spearman correlations of BMI with anthropometric measures and MNA score (N = 127).

Comparison	r_s_	P value
BMI vs. MUAC	+0.893	<0.001
BMI vs. CC	+0.885	<0.001
BMI vs. TSF	+0.967	<0.001
BMI vs. MAMC	+0.366	<0.001
BMI vs. MNA score	+0.885	<0.001

BMI, body mass index; MUAC, mid-upper arm circumference; CC, calf circumference; TSF, triceps skinfold thickness; MAMC, mid-arm muscle circumference; MNA, Mini Nutritional Assessment; r_s_, Spearman rank correlation coefficient. Positive values indicate direct correlations.

### 3.7. Correlation of BMI with biochemical parameters

In the full cohort, BMI was positively correlated with hemoglobin (r_s_=+0.320, P < 0.001), serum iron (r_s_=+0.408, P < 0.001), albumin (r_s_=+0.451, P < 0.001), and zinc (r_s_=+0.375, P < 0.001), but not with folate or vitamin B12 (Table 5). Most associations were no longer significant after stratification. The only significant subgroup correlations were a positive correlation between BMI and hemoglobin in remission (r_s_=+0.267, P = 0.033) and a weak inverse correlation between BMI and hemoglobin among participants with GLIM malnutrition (r_s_ = −0.267, P = 0.039).

**Table 5:** Spearman correlations of BMI with biochemical parameters, overall and by subgroup.

Comparison	Total(n = 127)	Active(n = 63)	Remission(n = 64)	Normal status(n = 67)	Malnutrition(n = 60)
BMI vs. hemoglobin	+0.320 (<0.001)	−0.090 (0.484)	+0.267 (0.033)	+0.172 (0.164)	−0.267 (0.039)
BMI vs. serum iron	+0.408 (<0.001)	+0.200 (0.115)	+0.027 (0.831)	−0.120 (0.335)	+0.213 (0.102)
BMI vs. folate	+0.060 (0.500)	−0.059 (0.649)	−0.019 (0.880)	+0.153 (0.216)	−0.082 (0.531)
BMI vs. vitamin B12	+0.014 (0.878)	+0.059 (0.646)	+0.228 (0.070)	+0.094 (0.447)	−0.052 (0.693)
BMI vs. albumin	+0.451 (<0.001)	+0.197 (0.122)	+0.135 (0.287)	−0.031 (0.804)	+0.173 (0.186)
BMI vs. zinc	+0.375 (<0.001)	+0.040 (0.756)	+0.144 (0.256)	+0.129 (0.299)	+0.048 (0.718)

Values are r_s_ (P value). BMI, body mass index; r_s_, Spearman rank correlation coefficient. Positive values indicate direct correlations and negative values indicate inverse correlations

## 4. Discussion

This cross-sectional study provides a multidimensional assessment of nutritional status in Bangladeshi adults with Crohn’s disease by integrating GLIM-based protein-energy malnutrition classification, anthropometry, the full Mini Nutritional Assessment (MNA), and selected biochemical markers. Nearly half of the participants met the study definition of GLIM malnutrition, and malnutrition was considerably more common among those with active disease. Active Crohn’s disease was also associated with consistently poorer anthropometric measurements and lower hemoglobin, serum iron, albumin, and zinc concentrations. BMI correlated closely with the other anthropometric measures and the MNA score, whereas its associations with biochemical indices were weaker and became mostly non-significant after subgroup stratification. The objective disease-activity sensitivity analysis showed a similar overall nutritional pattern across the different scoring systems, indicating that the observed differences were not explained by clinical symptoms alone in the absence of objective inflammatory activity. Together, these findings support a multidimensional assessment approach that combines evaluation of physical tissue depletion with assessment of micronutrient abnormalities and inflammation-related biochemical changes.

The observed frequency of malnutrition is broadly consistent with previous studies reporting malnutrition in approximately 20% to 80% of patients with Crohn’s disease, with a substantially higher burden during active disease than during remission [2,3]. The marked difference between the two disease phases in this study is also consistent with reports from India and Brazil, where active Crohn’s disease was associated with poorer nutritional status [17,22]. This pattern can be explained by the effects of inflammation in active Crohn’s disease, which can simultaneously reduce food intake, impair nutrient absorption, increase enteric nutrient loss, and intensify cytokine-mediated catabolism [4,5]. The smaller but clinically relevant proportion of malnutrition observed during remission indicates that nutritional recovery may lag behind symptomatic or structural disease control and supports continued surveillance during remission [5,24]. Extraintestinal manifestations were also more frequent in active disease, reinforcing the broader systemic burden accompanying inflammatory activity [25]. Although the cross-sectional design does not establish causality, the coexistence of active inflammation, poorer nutritional status, and a greater burden of systemic manifestations highlights the importance of nutritional assessment in Crohn’s disease. Importantly, malnutrition was also present in a substantial proportion of patients in remission, supporting the integration of routine nutritional assessment into ongoing CD care regardless of disease activity.

BMI, MUAC, CC, TSF, and MAMC were all lower in active disease, and BMI showed strong to very strong positive correlations with MUAC, CC, TSF, and MNA score, while its correlation with MAMC was more modest.. These findings suggest that BMI was a useful alternative for gross tissue depletion in this clinical setting and support the practical value of simple bedside anthropometry where advanced body-composition methods are unavailable. This role should, however, be separated from the assessment of micronutrient status. BMI may reflect broad changes in body mass and tissue stores, but it cannot directly represent dynamic iron, vitamin, or trace-element reserves. The present results therefore do not diminish the utility of BMI; rather, they define its appropriate role as an accessible entry-point measure that should be interpreted alongside other laboratory assessments for better evaluation of nutritional status [3,11]. The strong association between BMI and MNA also supports the internal consistency of the somatic assessment, although the MNA was developed mainly for older adults and was used here only as a complementary measure.

The South Asian thin-fat phenotype is particularly relevant to interpreting these findings. In this population, central adiposity and metabolic vulnerability may coexist with comparatively low body weight or reduced lean mass, allowing sarcopenia or tissue depletion to remain concealed behind an apparently acceptable BMI [14]. In the present Bangladeshi cohort, however, active Crohn’s disease was associated with parallel reductions in BMI, MUAC, CC, TSF, and MAMC. This pattern suggests that the combined metabolic demand, reduced intake, and gastrointestinal nutrient loss associated with active disease were sufficiently pronounced to affect both fat- and lean-tissue proxies. In the present study, GLIM classification was operationalised using low BMI (<18.5 kg/m²) as the phenotypic criterion because weight-loss history was incomplete and no prospectively specified, validated regional cutoff for reduced muscle mass was available. Consequently, participants with a normal BMI but reduced muscle mass may not have been identified as malnourished using this study-specific operational definition. This limitation reflects the available phenotypic measures in the present study rather than a limitation of the GLIM framework itself. GLIM is designed to identify protein-energy malnutrition and macronutritional wasting; it is not intended to diagnose isolated micronutrient or trace-element deficiencies. Accordingly, GLIM-based assessment should be complemented by targeted biochemical screening to identify micronutrient deficiencies that may occur independently of, or persist despite, relatively stable anthropometric findings.

The biochemical findings reflected both nutritional disturbance and inflammatory physiology. Hemoglobin, serum iron, albumin, and zinc were lower during active disease, whereas folate and vitamin B12 did not differ significantly between groups. These findings are consistent with previous reports of anemia, iron deficiency, hypoalbuminemia, and zinc deficiency in Crohn’s disease [17,26,27], although they should be interpreted with caution. Albumin and zinc are negative acute-phase reactants, while hemoglobin and iron may be altered by intestinal blood loss, inflammation, malabsorption, disease location, treatment exposure, and supplementation. Consequently, lower concentrations during active disease cannot be attributed solely to depleted nutrient stores. In a metabolically vulnerable South Asian population, hepatic lipid accumulation and insulin resistance may additionally influence hepatic handling of metabolic substrates [28]. However, hepatic fat and insulin resistance were not measured in the present study, and this mechanism should therefore be regarded only as contextual biological consideration rather than an explanation for the observed biochemical findings. The weak-to-moderate correlations between BMI and biochemical markers, together with the loss of most associations after subgrouping, confirm that anthropometry and biochemical testing provide complementary rather than interchangeable information [29,30]. The weak inverse correlation between BMI and hemoglobin among participants with GLIM malnutrition was counterintuitive and should be interpreted as exploratory. Restriction of BMI range within the subgroup, inflammation, hydration, blood loss, transfusion, treatment, and multiple testing may have contributed to this isolated association.

This study has several strengths. Disease activity was evaluated using complementary clinical, endoscopic, and radiological assessments, and the objective sensitivity analysis supported the primary nutritional comparisons. Nutritional evaluation incorporated multiple anthropometric measures, the MNA as an adjunct, GLIM-based classification, and biochemical testing. Complete data were available for all enrolled participants, and the study contributes evidence from a South Asian tertiary-care setting in which multidimensional nutritional data remain limited. Several limitations should also be acknowledged. The cross-sectional design precludes temporal or causal inference, and the single-center setting limits generalizability. The prespecified exclusion of diabetes mellitus, tuberculosis, unresolved tuberculosis-Crohn’s disease diagnostic uncertainty, and other systemic conditions was necessary to reduce confounding from disorders that independently cause weight loss, biochemical abnormalities, or systemic wasting, but it also produced a selected group of patients that may not represent the full tertiary-care Crohn’s disease population in Bangladesh. We relied on low BMI for GLIM classification because reliable prior weight history, direct body-composition assessment, and a validated regional muscle-mass cutoff were unavailable. The MNA is not specific to Crohn’s disease or to younger adults. Inflammatory markers such as C-reactive protein were not included, and treatment, supplementation, hydration, blood loss, or transfusion may have influenced biochemical results. Finally, the exploratory analyses involved multiple comparisons, and isolated subgroup findings should be interpreted cautiously.

These findings have practical implications for Crohn’s disease care. Nutritional assessment should not be restricted to overtly symptomatic or hospitalized patients, because protein-energy malnutrition and biochemical deficiencies may persist during remission. In resource-limited settings, BMI and simple circumference and skinfold measurements can provide useful information about gross tissue depletion, but they should be combined with targeted laboratory testing. Future multicenter longitudinal studies should incorporate prospectively recorded weight change, validated regional muscle-mass thresholds, direct body-composition techniques, adult-appropriate nutritional screening tools, inflammatory markers, standardized dietary assessment, and outcome-based follow-up. Such studies are needed to determine whether early identification and correction of nutritional impairment improve functional status, treatment response, and the long-term course of Crohn’s disease.

## Supporting information

S1 File STROBE checklist for cross-sectional studies. (DOCX)

S2 Data De-identified participant-level data underlying the analyses. (XLSX)
