# Supplementary material for: Multidimensional nutritional assessment in Crohn’s disease: Cross-sectional comparison of active disease and remission
Source: PLoS One. 2026 Sep 3;21(9):e0352372. doi: 10.1371/journal.pone.0352372 (PMC13541178; doi:10.1371/journal.pone.0352372)
Supplement: Supplementary file 1 [file pone.0352372.s001.docx]

**STROBE Checklist**

**Multidimensional nutritional assessment in Crohn’s disease: cross-sectional comparison of active disease and remission**

| **Section/topic** | **Item** | **STROBE recommendation** | **Where reported in revised manuscript** | **Status** |
| --- | --- | --- | --- | --- |
| Title and abstract | 1(a) | Indicate the study’s design with a commonly used term in the title or the abstract. | Title (p. 1); unstructured Abstract (p. 2). The title identifies the study as cross-sectional. | **Reported** |
| Title and abstract | 1(b) | Provide in the abstract an informative and balanced summary of what was done and what was found. | Unstructured Abstract (pp. 2-3): summarizes the setting, participants, disease-activity assessment, nutritional assessment, principal results, and interpretation. | **Reported** |
| Introduction | 2 | Explain the scientific background and rationale for the investigation being reported. | Introduction (pp. 4-6): scientific background, nutritional burden in Crohn's disease, South Asian context, and rationale for multidimensional assessment. | **Reported** |
| Introduction | 3 | State specific objectives, including any prespecified hypotheses. | End of Introduction (p. 6): specific study aim and objective disease-activity sensitivity analysis are stated. | **Reported** |
| Methods | 4 | Present key elements of study design early in the paper. | Abstract (p. 2); Study Design and Participants (pp. 7-8): cross-sectional observational design is stated early. | **Reported** |
| Methods | 5 | Describe the setting, locations, and relevant dates, including periods of recruitment, exposure, follow-up, and data collection. | Study Design and Participants (p. 7): tertiary-care setting in Dhaka, Bangladesh, and recruitment period (1 February 2024 to 31 January 2025) are reported. Follow-up is not applicable to the cross-sectional design. | **Reported** |
| Methods | 6(a) | Give the eligibility criteria, and the sources and methods of selection of participants. | Study Design and Participants (pp. 7-8): age eligibility, Crohn's disease diagnosis, consecutive assessment, exclusion criteria, and final inclusion are described. | **Reported** |
| Methods | 7 | Clearly define all outcomes, exposures, predictors, potential confounders, and effect modifiers. Give diagnostic criteria, if applicable. | Disease Activity Assessment (p. 8); Nutritional Assessment (p. 9); Definition of Malnutrition (pp. 9-10); Biochemical and Imaging Assessment (p. 10). | **Reported** |
| Methods | 8* | For each variable of interest, give sources of data and details of methods of assessment (measurement). Describe comparability of assessment methods if there is more than one group. | Nutritional Assessment (p. 9) and Biochemical and Imaging Assessment (p. 10): measurement methods, equipment, units, and calculation of MAMC are described; the same assessment approach was used in both disease-activity groups. | **Reported** |
| Methods | 9 | Describe any efforts to address potential sources of bias. | Study Design and Participants (pp. 7-8), Disease Activity Assessment (p. 8), Nutritional Assessment (p. 9), and Statistical Analysis (p. 11): consecutive sampling, prespecified exclusions, standardized measurements, multimodal disease-activity assessment, objective sensitivity analysis, complete-case analysis, and source-record checks are described. | **Reported** |
| Methods | 10 | Explain how the study size was arrived at. | Sample Size (p. 10): source study, formula, assumptions, power, and achieved sample size are reported. | **Reported** |
| Methods | 11 | Explain how quantitative variables were handled in the analyses. If applicable, describe which groupings were chosen and why. | Nutritional Assessment and Definition of Malnutrition (p. 9); Statistical Analysis (p. 11): quantitative variables, GLIM BMI threshold, MNA categories, non-parametric handling, and subgrouping are described. | **Reported** |
| Methods | 12(a) | Describe all statistical methods, including those used to control for confounding. | Statistical Analysis (p. 11): chi-square, Student's t-test, Mann-Whitney U test, Pearson/Spearman correlations, agreement statistics, significance threshold, and multiplicity approach are specified. Analyses are unadjusted; no confounder-adjusted model is reported. | **Partly reported** |
| Methods | 12(b) | Describe any methods used to examine subgroups and interactions. | Statistical Analysis (p. 11); disease-activity sensitivity/severity analyses (pp. 15-17) and subgroup correlations (pp. 22-23) are described and reported. | **Reported** |
| Methods | 12(c) | Explain how missing data were addressed. | Study Design and Participants (p. 8) states that no missing observations were identified; Statistical Analysis (p. 11) states that complete observations were used and no data were imputed. | **Reported** |
| Methods | 12(d) | If applicable, describe analytical methods taking account of sampling strategy. | Not applicable: consecutive sampling was used, with no complex sampling design requiring weighting, clustering, or stratification adjustment. | **Not applicable** |
| Methods | 12(e) | Describe any sensitivity analyses. | Disease Activity Assessment (p. 8) defines the objective sensitivity analysis; results and agreement statistics are reported in Results (pp. 15-16) and Figure 2 (p. 17). | **Reported** |
| Results | 13(a) | Report numbers of individuals at each stage of study—eg numbers potentially eligible, examined for eligibility, confirmed eligible, included in the study, completing follow-up, and analysed. | Participant Flow and Baseline Characteristics (pp. 12-13; Figure 1): 185 assessed, 58 excluded, 127 included/analyzed, with 63 active and 64 in remission. | **Reported** |
| Results | 13(b) | Give reasons for non-participation at each stage. | Figure 1 and its caption (pp. 12-13) provide the reasons and counts for exclusions, including tuberculosis, diabetes mellitus, thyroid disorders, malignancy, other specified conditions, and TB-Crohn's diagnostic uncertainty. | **Reported** |
| Results | 13(c) | Consider use of a flow diagram. | Participant flow diagram provided as Figure 1 (pp. 12-13). | **Reported** |
| Results | 14(a) | Give characteristics of study participants (eg demographic, clinical, social) and information on exposures and potential confounders. | Baseline characteristics are reported in Results/Table 1 (pp. 13-15); anthropometric and biochemical characteristics are presented in Results/Tables 2-3 (pp. 17-20). | **Reported** |
| Results | 14(b) | Indicate number of participants with missing data for each variable of interest. | Study Design and Participants (p. 8) and Results (p. 12) state that complete data were available for all variables; no missing observations were identified. | **Reported** |
| Results | 15* | Report numbers of outcome events or summary measures. | Outcome and summary measures are reported in Results (pp. 15-23), including disease-activity agreement, anthropometry/MNA, GLIM classification, biochemical parameters, and correlation analyses. | **Reported** |
| Results | 16(a) | Give unadjusted estimates and, if applicable, confounder-adjusted estimates and their precision (eg, 95% confidence interval). Make clear which confounders were adjusted for and why they were included. | Results/Tables 1-5 and Figures 2-4 (pp. 13-23) report unadjusted frequencies, medians/IQRs, P values, correlation coefficients, and agreement statistics. Confidence intervals and adjusted estimates are not reported. | **Partly reported** |
| Results | 16(b) | Report category boundaries when continuous variables were categorized. | MNA category boundaries and GLIM low-BMI threshold are stated in Methods (p. 9). Categorical biochemical low/normal results are presented in Figure 4, but the laboratory cutoff values are not explicitly listed in the manuscript. | **Partly reported** |
| Results | 16(c) | If relevant, consider translating estimates of relative risk into absolute risk for a meaningful time period. | Not applicable: relative-risk estimates were not calculated or reported in this cross-sectional descriptive/comparative analysis. | **Not applicable** |
| Results | 17 | Report other analyses done—eg analyses of subgroups and interactions, and sensitivity analyses. | Other analyses include objective disease-activity sensitivity and severity comparisons (pp. 15-17) and disease-activity/nutritional-status subgroup correlations (pp. 22-23). | **Reported** |
| Discussion | 18 | Summarise key results with reference to study objectives. | Opening Discussion (pp. 23-24) summarizes the principal findings in relation to the study objective. | **Reported** |
| Discussion | 19 | Discuss limitations of the study, taking into account sources of potential bias or imprecision. Discuss both direction and magnitude of any potential bias. | Limitations are discussed on p. 27, including cross-sectional design, single-center setting, selected exclusions, GLIM operationalization, lack of direct body-composition and inflammatory-marker data, MNA limitations, treatment/supplementation effects, and multiple comparisons. Direction/magnitude of potential bias are not quantified. | **Partly reported** |
| Discussion | 20 | Give a cautious overall interpretation of results considering objectives, limitations, multiplicity of analyses, results from similar studies, and other relevant evidence. | Discussion (pp. 23-28) interprets the findings in relation to study objectives, prior evidence, biological context, limitations, and exploratory multiple comparisons. | **Reported** |
| Discussion | 21 | Discuss the generalisability (external validity) of the study results. | Generalisability is addressed in Study Design and Participants (p. 8) and Discussion limitations (p. 27), noting the selected single-center tertiary-care Bangladeshi cohort. | **Reported** |
| Other information | 22 | Give the source of funding and the role of the funders for the present study and, if applicable, for the original study on which the present article is based. | Financial Disclosure section of the PLOS ONE online submission system; funding source and funder role reported. | **Reported** |
