# Supplementary material for: Multidimensional nutritional assessment in Crohn’s disease: Cross-sectional comparison of active disease and remission
Source: PLoS One. 2026 Sep 3;21(9):e0352372. doi: 10.1371/journal.pone.0352372 (PMC13541178; doi:10.1371/journal.pone.0352372)
Supplement: Supplementary file 2 [file pone.0352372.s004.docx]

**Manuscript ID: PONE-D-26-28588**

**“Multidimensional nutritional assessment in Crohn’s disease: cross-sectional comparison of active disease and remission”**

Dear Dr. Marwan Salih Al-Nimer and Reviewer #1,

We sincerely thank you for the careful evaluation of our manuscript and for the detailed, constructive comments. We have revised the manuscript extensively in response to the journal requirements, the Academic Editor’s statistical comments, and Reviewer #1’s methodological and interpretive concerns. The revised manuscript now provides clearer statistical reporting, a more explicit South Asian context, a transparent disease-activity sensitivity analysis, a participant flow diagram with exclusion counts, a clarified GLIM operational definition, revised interpretation of biochemical markers, and additional visual summaries of the principal findings. We have also provided a completed STROBE checklist and deidentified participant-level data as Supporting Information.

Reviewer and editor comments are reproduced below in bold, followed by our responses. Locations refer to the revised manuscript.

# A. PLOS ONE Journal Requirements

1. Please ensure that your manuscript meets PLOS ONE’s style requirements, including those for file naming.

**Response:** We have revised the manuscript formatting and organization for resubmission and will upload the required files using the journal’s requested designations, including a clean unmarked manuscript, a marked-up revised manuscript, a Response to Reviewers file, and the Supporting Information files.

**Location/change:** Relevant revisions are reflected throughout the manuscript and in the resubmission file set.

2. PLOS requires the data underlying the findings to be freely available unless an approved ethical/legal restriction applies.

**Response:** We have addressed this requirement by preparing a deidentified participant-level dataset containing the data underlying the reported analyses. This dataset is being supplied as Supporting Information (S2). Direct participant identifiers have been removed, while the analytical variables needed to reproduce the reported findings are retained.

**Location/change:** Supporting Information: S2 dataset; supporting-information captions are listed at the end of the manuscript.

3. Please include captions for Supporting Information files at the end of the manuscript and update in-text citations accordingly.

**Response:** We have added Supporting Information captions at the end of the revised manuscript. The STROBE checklist is identified as S1 Checklist and the deidentified analytical dataset as S2.

**Location/change:** End of manuscript, Supporting Material section (pp. 32–33).

4. Reviewer-recommended references should be evaluated for relevance.

**Response:** We evaluated the suggested publications and incorporated those that were relevant to the revised scientific framing, including references concerning high-fructose dietary exposure, alcohol use in IBD, diet-associated insulin resistance, and hepatic metabolic handling. The reference list and in-text citations were revised accordingly.

**Location/change:** Introduction (pp. 4–6), Discussion (pp. 25–26), References (pp. 28–32).

# B. Academic Editor – Statistical Analysis Comments

a. The authors used the test of normality, while they did not mention the variables that did not follow the normal distribution.

**Response:** We revised the Statistical Analysis section to identify the continuous variables that were analyzed non-parametrically. These include age, disease duration, BMI, MUAC, CC, TSF, MAMC, MNA, hemoglobin, serum iron, folate, vitamin B12, albumin, and zinc because non-normality was present in at least one comparison group or the variable was assessed in smaller sex-specific subgroups.

**Location/change:** Methods, Statistical Analysis (p. 11).

b. The authors mentioned SD/SE. The results are not expressed as mean ± SE.

**Response:** We removed the SD/SE ambiguity. The revised manuscript states that normally distributed continuous variables are summarized as mean ± standard deviation (SD), while non-normally distributed variables are summarized as median and interquartile range (IQR). Standard error is no longer presented as a reporting option.

**Location/change:** Methods, Statistical Analysis (p. 11).

c. The authors did not mention that some results are expressed as medians (IQR), which are mentioned in the tables.

**Response:** This has been corrected. The revised Statistical Analysis section explicitly states that non-normally distributed variables are reported as median and IQR.

**Location/change:** Methods, Statistical Analysis (p. 11); Results Tables 1–5.

d. Continuous normally distributed data are expressed as mean ± SD. Write the statistical analysis test.

**Response:** The revised Methods now specify the independent-samples Student’s t-test for normally distributed continuous variables and the Mann–Whitney U test for non-normally distributed variables. Between-group categorical comparisons are performed using the chi-square test.

**Location/change:** Methods, Statistical Analysis (p. 11).

e. The Spearman’s correlation factor: write positive [+] or inverse [-].

**Response:** We now explicitly state that positive correlation coefficients indicate direct associations and negative coefficients indicate inverse associations. Signs are shown in the correlation tables and in the Results narrative.

**Location/change:** Methods, Statistical Analysis (p. 11); Results, Tables 4–5 (pp. 21–23).

# C. Reviewer #1

## Abstract

The abstract should explicitly identify the geographic/ethnic context and setting of the cohort.

**Response:** We agree. The Abstract now identifies the study population as 127 South Asian adults with Crohn’s disease attending a tertiary-care center in Dhaka, Bangladesh. This makes the regional context and the principal rationale of the study clear from the Abstract itself.

**Location/change:** Abstract (pp. 2–3).

The abstract conclusion should not describe albumin and zinc simply as inconsistent nutritional markers because both are affected by the acute-phase response and systemic inflammation.

**Response:** We agree and revised the interpretation. The Abstract now states that biochemical abnormalities should be interpreted alongside disease activity because markers such as albumin and zinc may be influenced by systemic inflammation in addition to nutritional status. We therefore emphasize a multidimensional assessment combining anthropometric and biochemical information.

**Location/change:** Abstract conclusion (p. 3).

## Introduction

The justification for using MNA-SF in a younger cohort is weak; the manuscript should transparently clarify the tool used and its role.

**Response:** We appreciate this concern. The revised manuscript no longer presents the nutritional questionnaire as a resource-driven substitute for adult IBD screening tools. It now describes the full Mini Nutritional Assessment (MNA; 0–30 points) used in the study and explicitly states that, because the MNA was not developed specifically for younger adults with Crohn’s disease, it was interpreted only as a complementary assessment and not as the primary diagnostic criterion for malnutrition.

**Location/change:** Introduction (p. 5); Methods, Nutritional Assessment (p. 9).

Clarify the BMI threshold used in this South Asian cohort and the limitations of BMI for detecting sarcopenia/malnutrition.

**Response:** We revised the Introduction and Methods to state that BMI <18.5 kg/m² was used as the phenotypic GLIM criterion for low BMI. We also explicitly discuss the South Asian thin-fat phenotype and the inability of BMI to distinguish fat mass from lean mass. MUAC, CC, TSF, and MAMC were interpreted alongside BMI but were not used as formal GLIM diagnostic triggers because a prospectively specified, validated regional cutoff for reduced muscle mass was unavailable.

**Location/change:** Introduction (pp. 5–6); Methods, Definition of Malnutrition (pp. 9–10).

Add a sentence on Westernized dietary exposures, fructose, and alcohol.

**Response:** We incorporated this suggestion in a more cautious form appropriate to an observational study in which these exposures were not directly measured. The revised Introduction notes that Westernized dietary exposures may influence intestinal and metabolic inflammation, including evidence relating high-fructose intake to worsening experimental colitis and alcohol exposure to adverse IBD outcomes and metabolic dysfunction.

**Location/change:** Introduction (p. 4), with supporting references 6–8.

Remove the repetitive GLIM wording.

**Response:** We condensed the repeated text. GLIM is now introduced once as a standardized framework for diagnosing adult protein-energy malnutrition and is then linked directly to its application in Crohn’s disease.

**Location/change:** Introduction (p. 5).

## Methods

The primary disease-activity definition is asymmetric. Provide the exact CDAI-only versus objective activity breakdown and perform an objective-only sensitivity analysis.

**Response:** We addressed this directly. The revised Methods prespecify a sensitivity analysis defining activity strictly by objective evidence on SES-CD and/or MRE, irrespective of CDAI. In the revised Results, all 63 participants classified as active were active on CDAI, SES-CD, and MRE, while all 64 participants in remission were inactive on all three modalities. Thus, 63 participants were positive on both CDAI and the objective composite, 64 were negative on both, and there were no CDAI-only or objective-only active cases. The objective definition reproduced the primary grouping exactly (100% agreement; Cohen’s κ=1.000), and no participant was reclassified.

**Location/change:** Methods, Disease Activity Assessment (p. 8); Results, Disease-Activity Classification and Objective Sensitivity Analysis (pp. 15–17); Figure 2.

Report exactly how many patients were excluded for tuberculosis, diabetes, and other exclusion criteria, and discuss the resulting effect on generalizability.

**Response:** We added a participant flow diagram showing 185 patients assessed, 58 excluded, and 127 included. The exclusion breakdown is reported as: other specified conditions (n=3), malignancy (n=1), thyroid disorders (n=2), diabetes mellitus (n=7), tuberculosis (n=13), and unresolved TB–Crohn’s diagnostic dilemma (n=32). The Methods and Discussion now explain that these exclusions were intended to reduce confounding from other causes of wasting and biochemical abnormalities, while acknowledging that the selected cohort may be less representative of the full tertiary-care Crohn’s disease population.

**Location/change:** Methods, Study Design and Participants (pp. 7–8); Results, Figure 1 (pp. 12–13); Discussion limitations (p. 27).

Specify the cutoff used to define reduced muscle mass under GLIM.

**Response:** We clarified that reduced muscle mass was not used as a formal GLIM diagnostic trigger in this analysis because no prospectively specified, validated regional cutoff was available. Weight-loss history was also incomplete. Accordingly, the phenotypic criterion used for the study-specific GLIM classification was low BMI (<18.5 kg/m²), while the etiologic criterion was fulfilled by Crohn’s disease-related inflammatory and gastrointestinal burden.

**Location/change:** Methods, Definition of Malnutrition (pp. 9–10).

## Results

Resolve the discrepancy in the malnutrition subgroup correlation between BMI and hemoglobin.

**Response:** We rechecked the analysis and confirmed that the correlation is inverse: rₛ=-0.267, P=0.039. The negative sign is now reported consistently in the Results text and Table 5. Because this finding is counterintuitive, we also explicitly discuss it as exploratory and note possible influences including restricted BMI range, inflammation, hydration, blood loss, transfusion, treatment, and multiple testing.

**Location/change:** Results, Correlation of BMI With Biochemical Parameters (pp. 22–23); Discussion (pp. 26–27).

Replace the GLIM table and heavy biochemical categorical table with clearer visual summaries.

**Response:** We added the requested visual summaries. Figure 3 presents the prevalence of GLIM-defined malnutrition versus normal nutritional status in active disease and remission. Figure 4 presents the prevalence of low hemoglobin, serum iron, albumin, and zinc by disease-activity group.

**Location/change:** Results, Figures 3 and 4 (pp. 19 and 21).

## Discussion

Add a dedicated discussion of the South Asian thin-fat phenotype.

**Response:** We added a dedicated paragraph explaining that central adiposity and metabolic vulnerability may coexist with relatively low body weight or reduced lean mass in South Asian populations, potentially concealing tissue depletion behind an apparently acceptable BMI. We then relate this context to the parallel reductions in BMI, MUAC, CC, TSF, and MAMC observed in participants with active Crohn’s disease.

**Location/change:** Discussion (p. 25).

Do not criticize GLIM for failing to identify isolated micronutrient deficiencies.

**Response:** We agree. The revised Discussion now states that GLIM is designed to identify protein-energy malnutrition/macronutritional wasting and is not intended to diagnose isolated micronutrient or trace-element deficiencies. We therefore recommend complementing GLIM-based assessment with targeted biochemical screening when micronutrient deficiencies are clinically relevant.

**Location/change:** Discussion (pp. 25–26).

Smooth the transition between the clinical utility and limitations of BMI.

**Response:** We revised the anthropometry discussion to distinguish the role of BMI in detecting gross physical tissue depletion from its inability to represent dynamic micronutrient reserves. BMI is now presented as a practical entry-point measure that should be interpreted alongside other anthropometric and laboratory assessments rather than as a standalone indicator.

**Location/change:** Discussion (pp. 24–25).

Add context on hepatic metabolic state and the interpretation of albumin/metabolic markers.

**Response:** We incorporated the suggested metabolic context but deliberately used cautious language. The revised Discussion notes that hepatic lipid accumulation and insulin resistance may influence hepatic handling of metabolic substrates in a metabolically vulnerable population. We explicitly state that hepatic fat and insulin resistance were not measured and that this mechanism is contextual rather than an explanation for the observed biochemical findings.

**Location/change:** Discussion (p. 26), with reference 28.

## Additional revisions

**In addition to the requested changes, we have:** expanded the description of complete-data handling and source-record checks; clarified the MAMC calculation as MUAC (cm) − [π × TSF (mm) / 10]; incorporated a completed STROBE checklist; updated the reference list; and provided deidentified participant-level data as Supporting Information.

We appreciate the Editor’s and Reviewer’s thoughtful comments, which substantially improved the clarity, transparency, and interpretation of the manuscript. We hope that the revised version satisfactorily addresses all concerns and is now suitable for further consideration by PLOS ONE.

Sincerely,

Prodipta Chowdhury, on behalf of all authors

Corresponding Author

Department of Endocrinology, Bangladesh Medical University, Shahbagh, Dhaka, Bangladesh

E-mail: prodipta.r18@bmu.ac.bd; prodipta.ss38@gmail.com
