# Supplementary material for: Multidimensional nutritional assessment in Crohn’s disease: Cross-sectional comparison of active disease and remission
Source: PLoS One. 2026 Sep 3;21(9):e0352372. doi: 10.1371/journal.pone.0352372 (PMC13541178; doi:10.1371/journal.pone.0352372)
Supplement: Peer review history

# Peer review: Decision Letter 0

**DOI:** 10.1371/journal.pone.0352372.r001

**Authors:** Marwan Al-Nimer

4 Jul 2026

Dear Dr. Chowdhury,

Please submit your revised manuscript by Sep 02 2026 11:59PM. If you will need more time than this to complete your revisions, please reply to this message or contact the journal office at plosone@plos.org. When you're ready to submit your revision, log on to https://www.editorialmanager.com/pone/ and select the 'Submissions Needing Revision' folder to locate your manuscript file.

- A letter that responds to each point raised by the academic editor and reviewer(s). You should upload this letter as a separate file labeled 'Response to Reviewers'.
- A marked-up copy of your manuscript that highlights changes made to the original version. You should upload this as a separate file labeled 'Revised Manuscript with Track Changes'.
- An unmarked version of your revised paper without tracked changes. You should upload this as a separate file labeled 'Manuscript'.

If you would like to make changes to your financial disclosure, please include your updated statement in your cover letter. Guidelines for resubmitting your figure files are available below the reviewer comments at the end of this letter.

If applicable, we recommend that you deposit your laboratory protocols in protocols.io to enhance the reproducibility of your results. Protocols.io assigns your protocol its own identifier (DOI) so that it can be cited independently in the future. For instructions see: https://journals.plos.org/plosone/s/submission-guidelines#loc-laboratory-protocols. Additionally, PLOS ONE offers an option for publishing peer-reviewed Lab Protocol articles, which describe protocols hosted on protocols.io. Read more information on sharing protocols at https://plos.org/protocols?utm_medium=editorial-email&utm_source=authorletters&utm_campaign=protocols.

As the corresponding author, your ORCID iD is verified in the submission system and will appear in the published article. PLOS supports the use of ORCID, and we encourage all coauthors to register for an ORCID iD and use it as well. Please encourage your coauthors to verify their ORCID iD within the submission system before final acceptance, as unverified ORCID iDs will not appear in the published article. *Only*  the individual author can complete the verification step; PLOS staff *cannot*  verify ORCID iDs on behalf of authors.

We look forward to receiving your revised manuscript.

Kind regards,

Marwan Salih Al-Nimer, MD, PhD

Academic Editor

PLOS One

Journal Requirements:

When submitting your revision, we need you to address these additional requirements.

1.Please ensure that your manuscript meets PLOS ONE's style requirements, including those for file naming. The PLOS ONE style templates can be found at

https://journals.plos.org/plosone/s/file?id=wjVg/PLOSOne_formatting_sample_main_body.pdf and

https://journals.plos.org/plosone/s/file?id=ba62/PLOSOne_formatting_sample_title_authors_affiliations.pdf

2. In the online submission form, you indicated that “The dataset generated and/or analyzed during the current study is not publicly available because it contains potentially sensitive information, and public sharing could compromise participant confidentiality. De-identified data relevant to the findings of this study may be made available to qualified researchers upon reasonable request and subject to approval by the Institutional Review Board of Bangladesh Medical University, Shahbagh, Dhaka. Requests for data access should be directed to the Institutional Review Board at provc_rnd@bsmmu.edu.bd, referencing study registration number 4764 (approved on 11 January 2024). Data will be shared in accordance with participant consent, institutional policies, and applicable ethical and legal requirements.”

All PLOS journals now require all data underlying the findings described in their manuscript to be freely available to other researchers, either 1. In a public repository, 2. Within the manuscript itself, or 3. Uploaded as supplementary information.

This policy applies to all data except where public deposition would breach compliance with the protocol approved by your research ethics board. If your data cannot be made publicly available for ethical or legal reasons (e.g., public availability would compromise patient privacy), please explain your reasons on resubmission and your exemption request will be escalated for approval.

3. Please include captions for your Supporting Information files at the end of your manuscript, and update any in-text citations to match accordingly. Please see our Supporting Information guidelines for more information: http://journals.plos.org/plosone/s/supporting-information.

4. If the reviewer comments include a recommendation to cite specific previously published works, please review and evaluate these publications to determine whether they are relevant and should be cited. There is no requirement to cite these works unless the editor has indicated otherwise.

Additional Editor Comments:

**Major revision**

The section of the statistical analysis needs to be revised.

a. the authors used the test of normality, while they did not mention the variables that not follow the normal distribution

b. the authors mentioned SD/SE. The results are not expressed as mean +/- SE

c. In this section, the authors did not mention that some results are expressed as medians (IQR), which are mentioned in the tables.

d. The continuous normal distributed data expressed as mean +/- SD. Write the statistical analysis test

e. The Spearman's correlation factor (write positive [+] or inverse [-]

[Note: HTML markup is below. Please do not edit.]

Reviewer's Responses to Questions

**Comments to the Author**

1. Is the manuscript technically sound, and do the data support the conclusions?

Reviewer #1: Yes

**********

2. Has the statistical analysis been performed appropriately and rigorously?

Reviewer #1: I Don't Know

**********

3. Have the authors made all data underlying the findings in their manuscript fully available?

The PLOS Data policy

Reviewer #1: Yes

**********

4. Is the manuscript presented in an intelligible fashion and written in standard English?

Reviewer #1: Yes

**********

Reviewer #1: Dear authors, here some comments that can improve your manuscript further,

Abstract

It states that the study evaluates a "population with CD, predominantly characterized by metabolic phenotype." It makes absolutely no mention of the geographic location, ethnicity, or setting of the study. However, the introduction explicitly reveals that the core novelty and justification of the paper is that this is a South Asian cohort displaying a specific "thin-fat, metabolically high-risk phenotype" in a "resource-limited setting."

This is matter as leaving out the geographic and ethnic context in the abstract significantly reduces the paper's discoverability and fails to establish its primary scientific hook. A reader or reviewer looking at the abstract alone would have no idea why this metabolic phenotype is predominant or where this data comes from.

Therefore, please revise the abstract's methods sentence to explicitly declare the cohort's origin and setting.

Example: "This healthcare-based cross-sectional study included 127 South Asian adults with CD..."

- a big issue about the conclusion

Your abstract highlights that patients with active CD had significantly lower albumin and zinc (P < 0.001), concluding that biochemical markers are less consistent for nutritional assessment. This conclusion ignores a major physiological confounding factor: albumin and zinc are negative acute-phase reactants.

In active Crohn's disease, systemic inflammation (driven by cytokines like IL-6 and TNF-alpha) suppresses the hepatic synthesis of albumin and alters zinc compartmentalization independent of actual nutritional status or body composition depletion. Labeling these as purely "less consistent nutritional markers" without accounting for active systemic inflammation is a major biochemical oversight.

I suggest that How to the authors have to rephrase abstract's conclusion to reflect this biological reality. Instead of framing it as simple inconsistency, note that biochemical markers are heavily confounded by systemic disease activity, which mandates the use of independent anthropometric measures.

Introduction

- In lines 64–67, the authors state: "The Mini Nutritional Assessment-Short Form (MNA-SF), although originally developed and validated for older adults, may serve as a pragmatic screening step..." They justify choosing it because other IBD-specific tools like the IBD-NST or INTICO-2 "may be less feasible in lower-resource settings."

This justification is methodologically weak. The MNA-SF includes items heavily weighted toward geriatrics (e.g., neuropsychological problems like dementia/depression, mobility, and age-related weight loss). In contrast, the MUST (Malnutrition Universal Screening Tool) or NRS-2002 (Nutritional Risk Screening) are completely free, internationally accepted, simple bedside tools validated for general adult medical/surgical populations—including IBD. Claiming that MNA-SF was chosen over IBD-NST due to "low resources" is a false dichotomy when other general adult tools are widely available and far more appropriate than a geriatric tool.

Recommend for the authors, you have to firmly defend why MNA-SF was used over MUST or NRS-2002 in a cohort with a mean age likely well below 65. If this was a secondary analysis of an existing dataset where MNA-SF happened to be the data collected, they must state this transparently rather than trying to justify it as a pragmatic clinical choice for young/middle-aged adults.

- In the introduction, authors should clarify that standard Western BMI cutoffs underestimate sarcopenia/malnutrition in this population. They should explicitly state whether they used Asia-Pacific specific BMI cutoffs (<18.5 kg/m^2 for underweight, but lower thresholds for overweight/obesity) to operationalize the GLIM criteria. If they used standard Western cutoffs, their ability to capture this unique phenotype is compromised.

- As you know, increased consumption of a western diet with or without alcohol drinking has been suggested to contribute to the rising incidence of IBD. I recommend to add a sentence for the future readers about alcohol and fructose: You may place this sentence after line 46-48:

(Crucially, the ongoing adoption of a Westernized diet rich in refined sugars and additives serves as a dual threat; high dietary fructose intake has been shown to heavily disrupt the gut microbiota and exacerbate experimental colitis Ref. 1, while the concurrent consumption of alcohol—a frequent but problematic habit in IBD patients—substantially raises the risk of clinical relapse Ref. 2 and compounds systemic insulin resistance and tissue inflammation Ref. 3.)

Reference:

1. Nishiguchi R, Basu S, Staab HA, et al. Dietary interventions to prevent high-fructose diet–associated worsening of colitis and colitis-associated tumorigenesis in mice. Carcinogenesis. 2021;42(6):842–852. doi:10.1093/carcin/bgab007.

2. Ramos GP, Kane S. Alcohol use in patients with inflammatory bowel disease. Gastroenterol Hepatol (N Y). 2021;17(5):211–216.

3. Alwahsh SM, Dwyer BJ, Forbes S, van Thiel DH, Lewis PJS, Ramadori G. Insulin production and resistance in different models of diet-induced obesity and metabolic syndrome. Int J Mol Sci. 2017;18(2):285. doi:10.3390/ijms18020285.

Why this sentence is important? It explicitly connects your discussion of the "metabolic phenotype" and "thin-fat South Asian cohort" to specific, actionable modern dietary habits. By pointing out how high-fructose and alcohol intake worsen actual mucosal inflammation and driving systemic insulin resistance simultaneously, you give the reader a clear, biologically plausible reason to look at both the clinical state of the intestine and the metabolic phenotype together.

Minor comment:

Lines 67–71: There is a repetitive echo in the text.

Text: "...or a formal diagnostic framework [8]. In this context, the Global Leadership Initiative on Malnutrition (GLIM) has provided a standardized framework for diagnosing adult malnutrition. Since its introduction in 2019, the GLIM framework has provided a standardized approach to diagnosing adult malnutrition..."

To correct that, condense this to remove the near-identical sentences. (e.g., "In this context, the Global Leadership Initiative on Malnutrition (GLIM) framework, introduced in 2019, provides a standardized approach to diagnosing adult malnutrition across diverse populations [9].").

Methods

In section 3, you state: "Patients were categorized as having active disease if activity was present on any one of these assessments [CDAI, SES-CD, MRE]. Remission required the absence of activity across all three modalities." This asymmetrical definition introduces a massive classification bias that artificially inflates the active group and dilutes the remission group. Clinical symptoms (CDAI) in IBD often correlate poorly with objective mucosal inflammation (endoscopy/imaging) due to concurrent irritable bowel syndrome (IBS), small intestinal bacterial overgrowth (SIBO), or bile acid malabsorption...

You must provide the exact breakdown of how many patients were classified as "active" based solely on CDAI versus objective structural measures (SES-CD/MRE). To make the paper methodologically sound, you should report a sub-analysis where active disease is defined strictly by objective evidence of inflammation (endoscopy or imaging) to prove that your nutritional findings aren't driven by non-inflammatory functional symptoms.

- In section 2, you exclude a vast laundry list of conditions: "including... diabetes mellitus, tuberculosis, thyroid disorders... or malignancy."

In South Asia, Tuberculosis (TB) and Diabetes are highly prevalent. More importantly, intestinal tuberculosis is the primary differential diagnosis for Crohn's disease in Bangladesh, and the two are notoriously difficult to separate. Furthermore, chronic inflammation and corticosteroid use in CD frequently induce secondary diabetes/dysglycemia. By entirely excluding anyone with diabetes or a history of TB, you may have inadvertently excluded your most severe, chronically ill, or misdiagnosed CD cases. This creates a highly selective sample that no longer represents the real-world tertiary care CD population in Dhaka.

I recommend that authors must state exactly how many patients were excluded based on these criteria in a flow diagram. In the discussion, you will need to defend this by stating that while it reduces generalizability, it was strictly necessary to isolate CD-specific malnutrition from other systemic wasting diseases.

- Under section 5, you write: "...or reduced muscle mass inferred from standard anthropometric measures..." The GLIM requires you to specify exactly how muscle mass was categorized as low. You gathered MUAC, CC, TSF, and calculated MAMC, but you do not state what reference population or cutoffs (e.g., below the 5th or 10th percentile of an Asian reference population) were used to officially trigger a "reduced muscle mass" diagnosis.

Therefore, explicitly state the cutoff values or percentiles used for MAMC or Calf Circumference to define "reduced muscle mass" under the GLIM framework. (e.g., "...reduced muscle mass inferred from an MAMC below the 10th percentile of regional reference standards...").

Results

- Look closely at the malnutrition column (n=60) in Table 7 for BMI vs. Hemoglobin. In the text (line 257), you state the correlation coefficient is negative: rs=-0.267, P=0.039. However, inside Table 7 itself, it is written as positive: 0.267 with P=0.039.Why it matters: This is a major contradiction that a statistical reviewer will catch instantly. If it is truly negative (rs = -0.267), it means that within the malnourished group, as BMI drops, hemoglobin goes up (or vice versa)—which is biologically paradoxical and counter-intuitive for Crohn's disease. If it is positive, the text is wrong.

I recommend to double-check your SPSS output. If it is positive, remove the minus sign from line 257. If it is genuinely negative, you must highlight this anomaly in your Discussion (e.g., perhaps driven by hemoconcentration/dehydration in the lowest-BMI patients, or blood transfusions given exclusively to the most severely malnourished individuals).

- To improve scannability and impact, you should extract the heavy numbers from the text and tables and convert them into clean visual layouts.

Graph 1: The Nutritional Gap (Replacing Table 3)

Instead of a small, dry table showing the GLIM percentages, use a clustered bar chart to visually emphasize the massive disparity in malnutrition rates between the two clinical phases.

Y-axis (prevelence (%)), X-axis (malnourhsed vs normal in active disease or remission

Graph 2: The Micronutrient Drop-off (Replacing Table 5)

Table 5 is a massive wall of text showing "Normal vs. Low" categorical splits. Converting the most critical systemic biomarkers (Iron, Zinc, Albumin, and Hemoglobin) into a comparative chart highlights the structural depletion of nutrients caused by active disease.

Discussion

- In the background of this project, a primary justification for investigating this cohort was the unique "thin-fat," metabolically vulnerable phenotype characteristic of South Asian populations. This phenotype can mask significant tissue and muscle wasting behind a misleadingly stable or normal BMI. Yet, the Discussion entirely omits this concept.

So, insert a dedicated paragraph in your anthropometry discussion. Explain that while regional body composition trends often complicate the use of BMI due to hidden sarcopenic obesity or central adiposity, the profound metabolic demand and nutrient malabsorption of active Crohn's disease in this Bangladeshi cohort resulted in clear, parallel depletion of both lean and fat compartments.

- In lines 304–306, you state: "...a substantial proportion of CD patients categorized as normal by the GLIM criteria exhibited biochemical deficiencies, emphasizing that GLIM alone is insufficient as a marker of malnutrition in CD."

This is a conceptual error regarding what the Global Leadership Initiative on Malnutrition (GLIM) framework is designed to do. GLIM is explicitly built to diagnose protein-energy undernutrition (loss of body mass, weight, and muscle driven by etiologic factors like chronic inflammation). It was never intended to serve as an index for isolated micronutrient or trace element deficiencies (often called "hidden hunger"). Criticizing GLIM for missing a zinc or folate deficiency is a misunderstanding of the framework's diagnostic boundaries.

Here rephrasing of the sentence (This underscores that while the GLIM framework effectively identifies macro-nutritional wasting and protein-energy malnutrition, it must be paired with routine biochemical screening to capture independent micronutrient and trace element deficiencies that can persist even in patients with a stable physical phenotype)

- Paragraph 3 presents an internal contradiction that disrupts your narrative flow. In lines 294–297, you strongly validate the clinical utility of BMI as a simple, powerful tool for resource-limited clinics. Immediately in the next sentence (lines 298–299), you pivot sharply with: "Despite the strong correlations observed in this group, the study also showed that BMI alone is not sufficient." This rapid flip reads like stylistic whiplash.

I recommend you to smooth the transition by explicitly separating somatic tracking from micronutrient tracking. State that your data confirms BMI is a highly reliable proxy for gross physical tissue depletion in this environment, but acknowledge that its inherent blind spot is its inability to reflect dynamic micronutrient reserves or micronutrient status.

- After line 309, I suggest to highlight the role of the liver and albumin, sugar, ... : ...However, biochemical markers in CD require cautious interpretation, as albumin is a negative acute-phase reactant, and hemoglobin, iron, and zinc may be influenced by inflammation, blood loss, malabsorption, disease location, treatment exposure, and supplementation (lines 309–313). Add this (This complex biological interpretation is further compounded by the metabolic state of the liver itself; hepatic lipid accumulation—a key feature of the regional metabolic phenotype—fundamentally impairs the liver’s baseline capacity to clear vital metabolic and nutritional indices from the portal blood, a pathophysiological bottleneck that typically requires profound hepatic fat reduction to reverse Ref.: Alwahsh, S. M., & Ramadori, G. (2015). How does bariatric surgery improve type II diabetes? The neglected importance of the liver in clearing glucose and insulin from the portal blood. Journal of Obesity & Weight Loss Therapy, 5(5), 1–4. https://doi.org/10.4172/2165-7904.1000280). The weak-to-moderate overall correlations between BMI and these markers..."

**********

what does this mean?). If published, this will include your full peer review and any attached files.

If you choose “no”, your identity will remain anonymous but your review may still be made public.

**Do you want your identity to be public for this peer review?**  For information about this choice, including consent withdrawal, please see our Privacy Policy

Reviewer #1: No

**********

[NOTE: If reviewer comments were submitted as an attachment file, they will be attached to this email and accessible via the submission site. Please log into your account, locate the manuscript record, and check for the action link "View Attachments". If this link does not appear, there are no attachment files.]

To ensure your figures meet our technical requirements, please review our figure guidelines: https://journals.plos.org/plosone/s/figures

You may also use PLOS’s free figure tool, NAAS, to help you prepare publication quality figures: https://journals.plos.org/plosone/s/figures#loc-tools-for-figure-preparation.

NAAS will assess whether your figures meet our technical requirements by comparing each figure against our figure specifications.

# Author comment: Author response to Decision Letter 1

**DOI:** 10.1371/journal.pone.0352372.r002

19 Aug 2026

Dear Dr. Marwan Salih Al-Nimer and Reviewer #1,

We sincerely thank you for the careful evaluation of our manuscript and for the detailed, constructive comments. We have revised the manuscript extensively in response to the journal requirements, the Academic Editor’s statistical comments, and Reviewer #1’s methodological and interpretive concerns. The revised manuscript now provides clearer statistical reporting, a more explicit South Asian context, a transparent disease-activity sensitivity analysis, a participant flow diagram with exclusion counts, a clarified GLIM operational definition, revised interpretation of biochemical markers, and additional visual summaries of the principal findings. We have also provided a completed STROBE checklist and deidentified participant-level data as Supporting Information.

Reviewer and editor comments are reproduced below in bold, followed by our responses. Locations refer to the revised manuscript.

A. PLOS ONE Journal Requirements

1. Please ensure that your manuscript meets PLOS ONE’s style requirements, including those for file naming.

Response: We have revised the manuscript formatting and organization for resubmission and will upload the required files using the journal’s requested designations, including a clean unmarked manuscript, a marked-up revised manuscript, a Response to Reviewers file, and the Supporting Information files.

Location/change: Relevant revisions are reflected throughout the manuscript and in the resubmission file set.

2. PLOS requires the data underlying the findings to be freely available unless an approved ethical/legal restriction applies.

Response: We have addressed this requirement by preparing a deidentified participant-level dataset containing the data underlying the reported analyses. This dataset is being supplied as Supporting Information (S2). Direct participant identifiers have been removed, while the analytical variables needed to reproduce the reported findings are retained.

Location/change: Supporting Information: S2 dataset; supporting-information captions are listed at the end of the manuscript.

3. Please include captions for Supporting Information files at the end of the manuscript and update in-text citations accordingly.

Response: We have added Supporting Information captions at the end of the revised manuscript. The STROBE checklist is identified as S1 Checklist and the deidentified analytical dataset as S2.

Location/change: End of manuscript, Supporting Material section (pp. 32–33).

4. Reviewer-recommended references should be evaluated for relevance.

Response: We evaluated the suggested publications and incorporated those that were relevant to the revised scientific framing, including references concerning high-fructose dietary exposure, alcohol use in IBD, diet-associated insulin resistance, and hepatic metabolic handling. The reference list and in-text citations were revised accordingly.

Location/change: Introduction (pp. 4–6), Discussion (pp. 25–26), References (pp. 28–32).

B. Academic Editor – Statistical Analysis Comments

a. The authors used the test of normality, while they did not mention the variables that did not follow the normal distribution.

Response: We revised the Statistical Analysis section to identify the continuous variables that were analyzed non-parametrically. These include age, disease duration, BMI, MUAC, CC, TSF, MAMC, MNA, hemoglobin, serum iron, folate, vitamin B12, albumin, and zinc because non-normality was present in at least one comparison group or the variable was assessed in smaller sex-specific subgroups.

Location/change: Methods, Statistical Analysis (p. 11).

b. The authors mentioned SD/SE. The results are not expressed as mean ± SE.

Response: We removed the SD/SE ambiguity. The revised manuscript states that normally distributed continuous variables are summarized as mean ± standard deviation (SD), while non-normally distributed variables are summarized as median and interquartile range (IQR). Standard error is no longer presented as a reporting option.

Location/change: Methods, Statistical Analysis (p. 11).

c. The authors did not mention that some results are expressed as medians (IQR), which are mentioned in the tables.

Response: This has been corrected. The revised Statistical Analysis section explicitly states that non-normally distributed variables are reported as median and IQR.

Location/change: Methods, Statistical Analysis (p. 11); Results Tables 1–5.

d. Continuous normally distributed data are expressed as mean ± SD. Write the statistical analysis test.

Response: The revised Methods now specify the independent-samples Student’s t-test for normally distributed continuous variables and the Mann–Whitney U test for non-normally distributed variables. Between-group categorical comparisons are performed using the chi-square test.

Location/change: Methods, Statistical Analysis (p. 11).

e. The Spearman’s correlation factor: write positive [+] or inverse [-].

Response: We now explicitly state that positive correlation coefficients indicate direct associations and negative coefficients indicate inverse associations. Signs are shown in the correlation tables and in the Results narrative.

Location/change: Methods, Statistical Analysis (p. 11); Results, Tables 4–5 (pp. 21–23).

C. Reviewer #1

Abstract

The abstract should explicitly identify the geographic/ethnic context and setting of the cohort.

Response: We agree. The Abstract now identifies the study population as 127 South Asian adults with Crohn’s disease attending a tertiary-care center in Dhaka, Bangladesh. This makes the regional context and the principal rationale of the study clear from the Abstract itself.

Location/change: Abstract (pp. 2–3).

The abstract conclusion should not describe albumin and zinc simply as inconsistent nutritional markers because both are affected by the acute-phase response and systemic inflammation.

Response: We agree and revised the interpretation. The Abstract now states that biochemical abnormalities should be interpreted alongside disease activity because markers such as albumin and zinc may be influenced by systemic inflammation in addition to nutritional status. We therefore emphasize a multidimensional assessment combining anthropometric and biochemical information.

Location/change: Abstract conclusion (p. 3).

Introduction

The justification for using MNA-SF in a younger cohort is weak; the manuscript should transparently clarify the tool used and its role.

Response: We appreciate this concern. The revised manuscript no longer presents the nutritional questionnaire as a resource-driven substitute for adult IBD screening tools. It now describes the full Mini Nutritional Assessment (MNA; 0–30 points) used in the study and explicitly states that, because the MNA was not developed specifically for younger adults with Crohn’s disease, it was interpreted only as a complementary assessment and not as the primary diagnostic criterion for malnutrition.

Location/change: Introduction (p. 5); Methods, Nutritional Assessment (p. 9).

Clarify the BMI threshold used in this South Asian cohort and the limitations of BMI for detecting sarcopenia/malnutrition.

Response: We revised the Introduction and Methods to state that BMI <18.5 kg/m² was used as the phenotypic GLIM criterion for low BMI. We also explicitly discuss the South Asian thin-fat phenotype and the inability of BMI to distinguish fat mass from lean mass. MUAC, CC, TSF, and MAMC were interpreted alongside BMI but were not used as formal GLIM diagnostic triggers because a prospectively specified, validated regional cutoff for reduced muscle mass was unavailable.

Location/change: Introduction (pp. 5–6); Methods, Definition of Malnutrition (pp. 9–10).

Add a sentence on Westernized dietary exposures, fructose, and alcohol.

Response: We incorporated this suggestion in a more cautious form appropriate to an observational study in which these exposures were not directly measured. The revised Introduction notes that Westernized dietary exposures may influence intestinal and metabolic inflammation, including evidence relating high-fructose intake to worsening experimental colitis and alcohol exposure to adverse IBD outcomes and metabolic dysfunction.

Location/change: Introduction (p. 4), with supporting references 6–8.

Remove the repetitive GLIM wording.

Response: We condensed the repeated text. GLIM is now introduced once as a standardized framework for diagnosing adult protein-energy malnutrition and is then linked directly to its application in Crohn’s disease.

Location/change: Introduction (p. 5).

Methods

The primary disease-activity definition is asymmetric. Provide the exact CDAI-only versus objective activity breakdown and perform an objective-only sensitivity analysis.

Response: We addressed this directly. The revised Methods prespecify a sensitivity analysis defining activity strictly by objective evidence on SES-CD and/or MRE, irrespective of CDAI. In the revised Results, all 63 participants classified as active were active on CDAI, SES-CD, and MRE, while all 64 participants in remission were inactive on all three modalities. Thus, 63 participants were positive on both CDAI and the objective composite, 64 were negative on both, and there were no CDAI-only or objective-only active cases. The objective definition reproduced the primary grouping exactly (100% agreement; Cohen’s κ=1.000), and no participant was reclassified.

Location/change: Methods, Disease Activity Assessment (p. 8); Results, Disease-Activity Classification and Objective Sensitivity Analysis (pp. 15–17); Figure 2.

Report exactly how many patients were excluded for tuberculosis, diabetes, and other exclusion criteria, and discuss the resulting effect on generalizability.

Response: We added a participant flow diagram showing 185 patients assessed, 58 excluded, and 127 included. The exclusion breakdown is reported as: other specified conditions (n=3), malignancy (n=1), thyroid disorders (n=2), diabetes mellitus (n=7), tuberculosis (n=13), and unresolved TB–Crohn’s diagnostic dilemma (n=32). The Methods and Discussion now explain that these exclusions were intended to reduce confounding from other causes of wasting and biochemical abnormalities, while acknowledging that the selected cohort may be less representative of the full tertiary-care Crohn’s disease population.

Location/change: Methods, Study Design and Participants (pp. 7–8); Results, Figure 1 (pp. 12–13); Discussion limitations (p. 27).

Specify the cutoff used to define reduced muscle mass under GLIM.

Response: We clarified that reduced muscle mass was not used as a formal GLIM diagnostic trigger in this analysis because no prospectively specified, validated regional cutoff was available. Weight-loss history was also incomplete. Accordingly, the phenotypic criterion used for the study-specific GLIM classification was low BMI (<18.5 kg/m²), while the etiologic criterion was fulfilled by Crohn’s disease-related inflammatory and gastrointestinal burden.

Location/change: Methods, Definition of Malnutrition (pp. 9–10).

Results

Resolve the discrepancy in the malnutrition subgroup correlation between BMI and hemoglobin.

Response: We rechecked the analysis and confirmed that the correlation is inverse: rs=-0.267, P=0.039. The negative sign is now reported consistently in the Results text and Table 5. Because this finding is counterintuitive, we also explicitly discuss it as exploratory and note possible influences including restricted BMI range, inflammation, hydration, blood loss, transfusion, treatment, and multiple testing.

Location/change: Results, Correlation of BMI With Biochemical Parameters (pp. 22–23); Discussion (pp. 26–27).

Replace the GLIM table and heavy biochemical categorical table with clearer visual summaries.

Response: We added the requested visual summaries. Figure 3 presents the prevalence of GLIM-defined malnutrition versus normal nutritional status in active disease and remission. Figure 4 presents the prevalence of low hemoglobin, serum iron, albumin, and zinc by disease-activity group.

Location/change: Results, Figures 3 and 4 (pp. 19 and 21).

Discussion

Add a dedicated discussion of the South Asian thin-fat phenotype.

Response: We added a dedicated paragraph explaining that central adiposity and metabolic vulnerability may coexist with relatively low body weight or reduced lean mass in South Asian populations, potentially concealing tissue depletion behind an apparently acceptable BMI. We then relate this context to the parallel reductions in BMI, MUAC, CC, TSF, and MAMC observed in participants with active Crohn’s disease.

Location/change: Discussion (p. 25).

Do not criticize GLIM for failing to identify isolated micronutrient deficiencies.

Response: We agree. The revised Discussion now states that GLIM is designed to identify protein-energy malnutrition/macronutritional wasting and is not intended to diagnose isolated micronutrient or trace-element deficiencies. We therefore recommend complementing GLIM-based assessment with targeted biochemical screening when micronutrient deficiencies are clinically relevant.

Location/change: Discussion (pp. 25–26).

Smooth the transition between the clinical utility and limitations of BMI.

Response: We revised the anthropometry discussion to distinguish the role of BMI in detecting gross physical tissue depletion from its inability to represent dynamic micronutrient reserves. BMI is now presented as a practical entry-point measure that should be interpreted alongside other anthropometric and laboratory assessments rather than as a standalone indicator.

Location/change: Discussion (pp. 24–25).

Add context on hepatic metabolic state and the interpretation of albumin/metabolic markers.

Response: We incorporated the suggested metabolic context but deliberately used cautious language. The revised Discussion notes that hepatic lipid accumulation and insulin resistance may influence hepatic handling of metabolic substrates in a metabolically vulnerable population. We explicitly state that hepatic fat and insulin resistance were not measured and that this mechanism is contextual rather than an explanation for the observed biochemical findings.

Location/change: Discussion (p. 26), with reference 28.

Additional revisions

In addition to the requested changes, we have: expanded the description of complete-data handling and source-record checks; clarified the MAMC calculation as MUAC (cm) − [π × TSF (mm) / 10]; incorporated a completed STROBE checklist; updated the reference list; and provided deidentified participant-level data as Supporting Information.

We appreciate the Editor’s and Reviewer’s thoughtful comments, which substantially improved the clarity, transparency, and interpretation of the manuscript. We hope that the revised version satisfactorily addresses all concerns and is now suitable for further consideration by PLOS ONE.

Attachment Submitted filename: Response to Reviewers.docx

# Editor report: Decision Letter 1

**DOI:** 10.1371/journal.pone.0352372.r003

**Authors:** Marwan Al-Nimer

23 Aug 2026

Multidimensional nutritional assessment in Crohn’s disease: cross-sectional comparison of active disease and remission

PONE-D-26-28588R1

Dear Dr. Prodipta Chowdhury,

We’re pleased to inform you that your manuscript has been judged scientifically suitable for publication and will be formally accepted for publication once it meets all outstanding technical requirements.

Within one week, you’ll receive an e-mail detailing the required amendments. When these have been addressed, you’ll receive a formal acceptance letter and your manuscript will be scheduled for publication.

An invoice will be generated when your article is formally accepted. Please note, if your institution has a publishing partnership with PLOS and your article meets the relevant criteria, all or part of your publication costs will be covered. Please make sure your user information is up-to-date by logging into Editorial Manager at Editorial Manager® and clicking the ‘Update My Information' link at the top of the page. For questions related to billing, please contact billing support.

If your institution or institutions have a press office, please notify them about your upcoming paper to help maximize its impact. If they’ll be preparing press materials, please inform our press team as soon as possible -- no later than 48 hours after receiving the formal acceptance. Your manuscript will remain under strict press embargo until 2 pm Eastern Time on the date of publication. For more information, please contact onepress@plos.org.

Kind regards,

Marwan Salih Al-Nimer, MD, PhD

Academic Editor

PLOS One

Additional Editor Comments (optional):

Reviewers' comments:

# Editor report: Acceptance letter

**DOI:** 10.1371/journal.pone.0352372.r004

**Authors:** Marwan Al-Nimer

PONE-D-26-28588R1

PLOS One

Dear Dr. Chowdhury,

I'm pleased to inform you that your manuscript has been deemed suitable for publication in PLOS One. Congratulations! Your manuscript is now being handed over to our production team.

At this stage, our production department will prepare your paper for publication. This includes ensuring the following:

* All references, tables, and figures are properly cited

* All relevant supporting information is included in the manuscript submission,

* There are no issues that prevent the paper from being properly typeset

You will receive further instructions from the production team, including instructions on how to review your proof when it is ready. Please keep in mind that we are working through a large volume of accepted articles, so please give us a few days to review your paper and let you know the next and final steps.

Lastly, if your institution or institutions have a press office, please let them know about your upcoming paper now to help maximize its impact. If they'll be preparing press materials, please inform our press team within the next 48 hours. Your manuscript will remain under strict press embargo until 2 pm Eastern Time on the date of publication. For more information, please contact onepress@plos.org.

You will receive an invoice from PLOS for your publication fee after your manuscript has reached the completed accept phase. If you receive an email requesting payment before acceptance or for any other service, this may be a phishing scheme. Learn how to identify phishing emails and protect your accounts at https://explore.plos.org/phishing.

If we can help with anything else, please email us at customercare@plos.org.

Thank you for submitting your work to PLOS One and supporting open access.

Kind regards,

PLOS One Editorial Office Staff

on behalf of

Professor Marwan Salih Al-Nimer

Academic Editor

PLOS One
